# Protocol for a Hybrid-type 1 pilot study of a randomized control trial of a brief, peer-delivered treatment to improve father depression and child mental health in Kenya

**DOI:** 10.1371/journal.pone.0325902

**Published:** 2025-06-26

**Authors:** Ali Giusto, Florence Jaguga, Dan Aburi, Mercy Korir, Winnie Maina, Wilter Rono, Michaela Greenlee

**Affiliations:** 1 Department of Psychology, Florida International University, Miami, Florida, United States of America; 2 Department of Alcohol and Drug Abuse Rehabilitation Services, Moi Teaching and Referral Hospital, Eldoret, Kenya; 3 Academic Model Providing Access to Healthcare, Eldoret, Kenya; 4 Department of Global Health & Population, Harvard T.H. Chan School of Public Health, Boston, Massachusetts, United States of America; Public Library of Science, UNITED STATES OF AMERICA

## Abstract

**Background:**

Few treatments specifically target father depression and alcohol use, despite their high prevalence worldwide and adverse impacts on families and youth. Fathers are also less likely to engage in treatment than female caregivers. To address this gap, a team of US- and Kenyan-based clinician-researchers developed *Learn, Engage, Act, Dedicate* (LEAD), a five-session, task-shifted psychosocial intervention for fathers in Eldoret, Kenya.

**Objective:**

This hybrid type-1 study aims to evaluate the feasibility, acceptability, and preliminary effectiveness of LEAD, a peer-father delivered psychosocial intervention for fathers at risk for depression and alcohol use. Secondary aims include exploring changes in child mental health and family functioning, potential mechanisms of change, and key implementation outcomes such as fidelity.

**Methods:**

We will conduct a hybrid type-1 pilot study using a parallel randomized controlled trial (RCT) design, enrolling 102 fathers randomized 2:1 to LEAD or a waitlist control group. All participants will be offered treatment as usual at baseline, with waitlist participants receiving LEAD following the waitlist period. Assessments will be conducted with fathers, their female partners, and one child aged 8–17. Primary aims are to explore changes in fathers’ depression and alcohol use; secondary aims include examining changes in family functioning and child well-being, understanding mechanisms driving change or nonresponse, and assessing the feasibility and acceptability of peer-father counselor implementation.

**Discussion:**

Findings will inform a future hypothesis-testing hybrid trial to examine LEAD’s effectiveness in improving father and child mental health and evaluate associated implementation strategies. This work will contribute to strategies for engaging and retaining men in mental health services.

**Trial Registration #:** NCT06489314 (ClinicalTrials.gov); July 4, 2024

## Introduction

Depression is the leading cause of disability worldwide, with approximately 13% of individuals in Kenya meeting criteria for depression [1,2]. Despite high prevalence, more than 75% of people in low- and middle-income countries (LMICs), including Kenya, do not receive treatment [3]. Treatment gaps are especially pronounced for men, who initiate and remain in care at rates four times lower than women [4,5]. Comorbid alcohol use, which disproportionately affects men [6,7], compounds the burden of depression. Improving mental health services for men is critical to reducing the global burden of depression and alcohol use, given their profound impact not only on men’s well-being but also on family functioning and child mental health (MH) [8].

Men’s depression and alcohol use influence child MH both directly [9,10] and indirectly through interparental conflict and impaired parenting [8,11,12]. Addressing fathers’ mental health is therefore central to strategies aimed at reducing the global burden of child MH problems, a leading cause of disability among youth [13]. Yet, fathers remain largely absent from MH treatment globally and in Kenya [3,14], due to barriers such as stigma, masculine norms [15], and alcohol use [16]. These factors exacerbate father mental health problems [17] and their consequences for families [18]. Consistent with the Family Stress Model [19], formative qualitative work in Kenya showed that economic strain was linked to depressive symptoms in men, leading to drinking, family conflict, and child MH difficulties [20]. Interventions for fathers must address these dynamics and consider ways to engage men [21].

To meet this need, Kenyan and US clinicians and researchers collaboratively designed Learn, Engage, Act, Dedicate (LEAD), a five-session behavioral activation (BA) intervention delivered by peer-father counselors [22]. LEAD integrates BA, which targets depressive behaviors [23,24]; motivational interviewing (MI), to enhance engagement and address alcohol use [25,26]; and masculinity discussions, to expand father identity beyond provider roles. In a proof-of-concept study using a randomized multiple baseline design with nine fathers and three peer-counselors [27], LEAD was acceptable, improved fathers’ depression and alcohol use, and enhanced parenting, interparental functioning, and child MH without adverse events.

### Objectives

Building on these promising results, this protocol describes a pilot hybrid type 1 effectiveness-implementation study using a randomized waitlist-control design [28,29]. Results will inform a fully powered RCT. Guided by community-engaged research principles and the RE-AIM framework [30], we will randomize 102 fathers (2:1) to LEAD or waitlist control, offering treatment as usual at baseline and LEAD to the waitlist after final assessment [31]. Recognizing that the study is not powered for definitive effectiveness, our pilot has three aims:

#### Aim 1: Explore the preliminary effectiveness of LEAD on father mental health.

The primary outcome is father depressive symptoms (PHQ-9), validated in Kenya. Secondary outcomes include fathers’ alcohol use, parenting, interparental problems, and child MH outcomes (ages 8–17) reported by fathers, co-caregivers, and children.

#### Aim 2: Explore mechanisms of change in father and child mental health outcomes.

Path analysis will test the pathway: LEAD ➔ positive activities ➔ father mental health ➔ parenting ➔ child MH. Mixed-methods interviews and LEAD session transcript analyses will explore patterns of change and non-change, using the Framework Method [31].

#### Aim 3: Explore feasibility and acceptability of implementing task-shifted mental health treatment for fathers in a low-resource setting.

A mixed-methods process evaluation will assess implementation outcomes, including reach, retention, fidelity, feasibility, acceptability, and barriers to/facilitators of scale-up and sustainability.

## Methods

### Trial design and guiding outcomes framework

**Fig 1:**
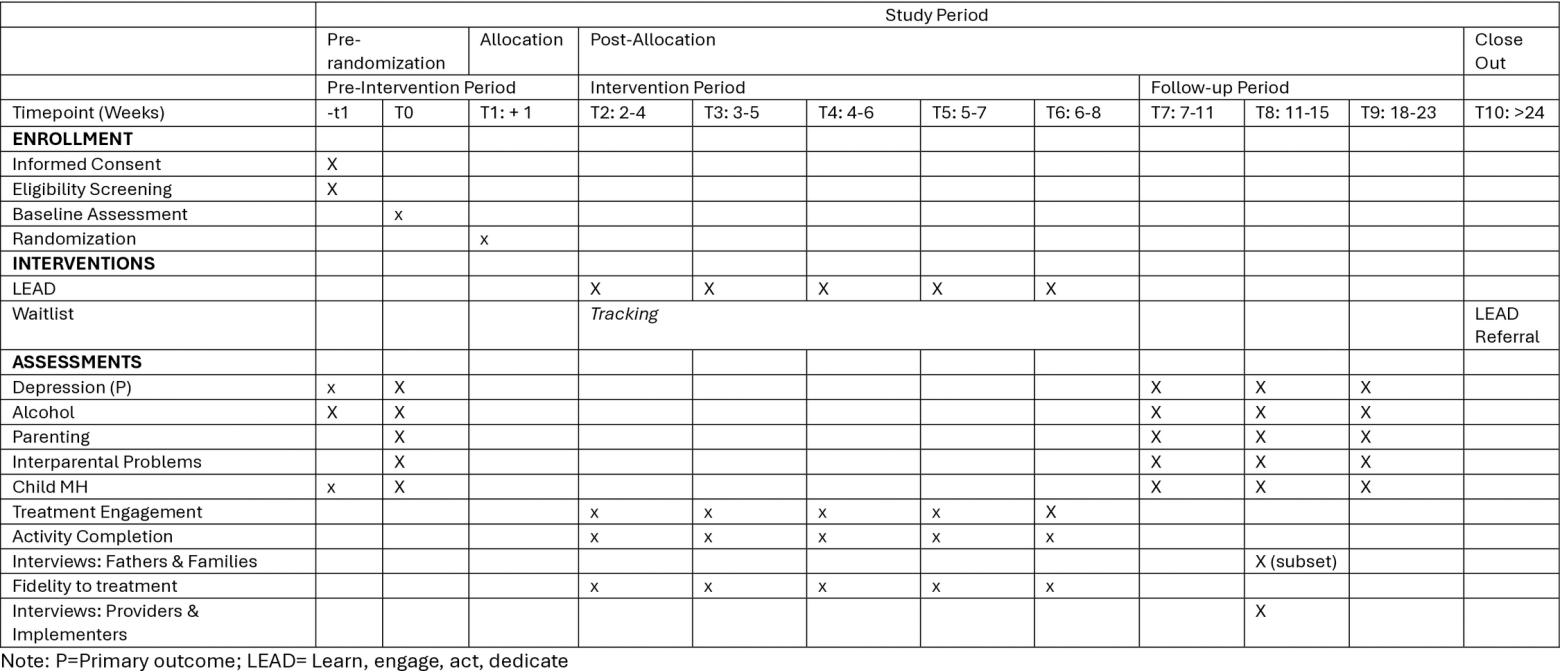
SPIRIT Schedule of Enrollments, Intervention, and Assessment.

**Fig 2:**
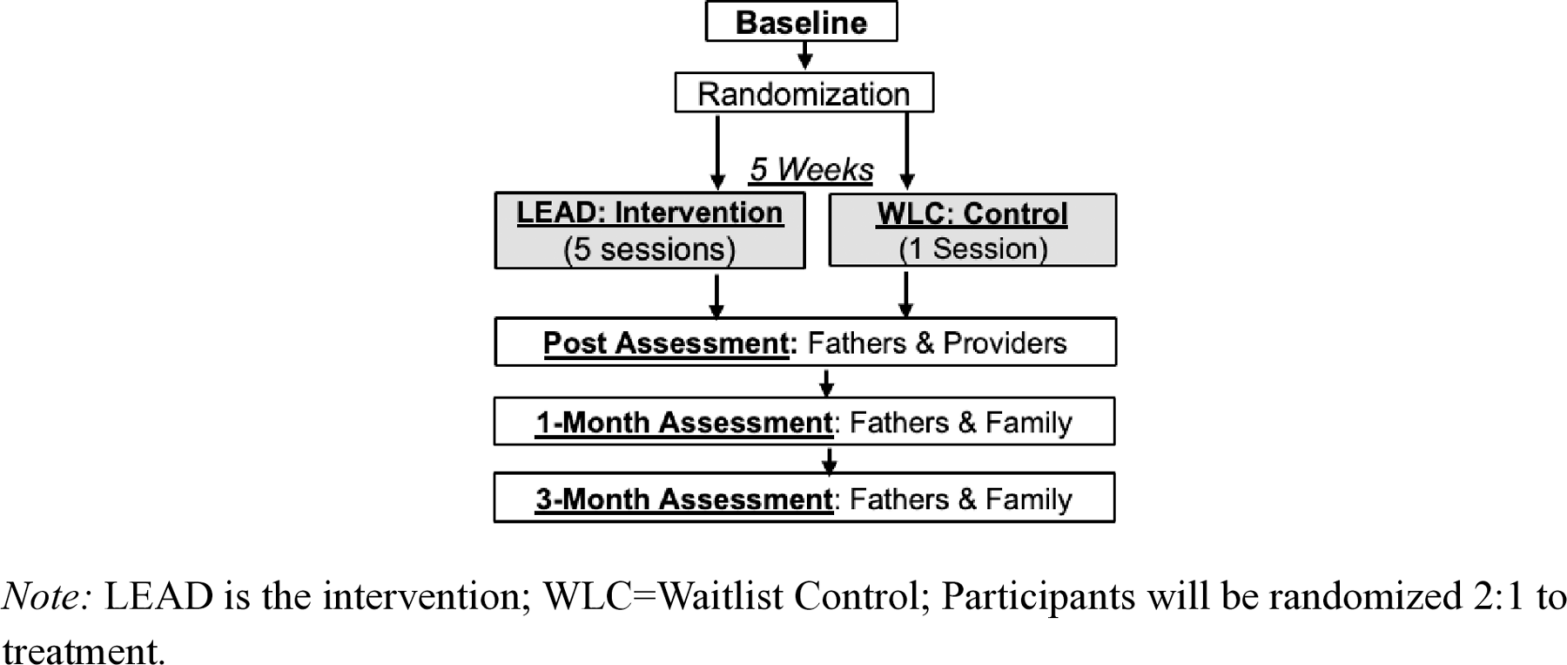
Study Design.

This study is a parallel-group, hybrid type 1 pilot randomized controlled trial (RCT) designed to evaluate the feasibility, acceptability, and preliminary effectiveness of LEAD in Eldoret, Kenya. Fathers (n = 102) will be randomized 2:1 to LEAD or a waitlist control group (Fig 1 for Schedule, Fig 2 Design). Randomization will be stratified by drinking severity using a random number generator to ensure group balance by a study consultant (not involved in on the ground study tasks). A 2:1 allocation ratio was chosen to maximize the number of participants receiving the intervention during the pilot phase and to increase service provision in a resource-limited setting. All participants will receive refferal to treatment as usual at baseline, and waitlist participants will be offered LEAD after completing the final assessment.

Assessments will occur at baseline, post-intervention, one-month, and three-month follow-ups, with data collected from fathers, co-caregivers, and children aged 8–17 years. Outcome assessors will be blinded to group assignment to minimize bias.

The hybrid type 1 design enables simultaneous evaluation of clinical outcomes (e.g., depression, alcohol use, family functioning) and key implementation outcomes (e.g., feasibility, acceptability, fidelity, reach). Findings will inform a future fully powered hybrid effectiveness-implementation trial.

### RE-AIM: clinical and implementation outcome guidance (Fig 3)

The Reach, Effectiveness/ Efficacy, Adoption, Implementation, and Maintenance (RE-AIM) framework guides outcome measurement in this hybrid effectiveness-implementation trial. We will employ an extension of RE-AIM that considers sustainability in each dimension [32]. We chose RE-AIM to guide the study given its focus on clinical and implementation outcomes, inclusion of patient- and provider-level outcomes, and prior application in Sub-Saharan Africa [33].

**Fig 3:**
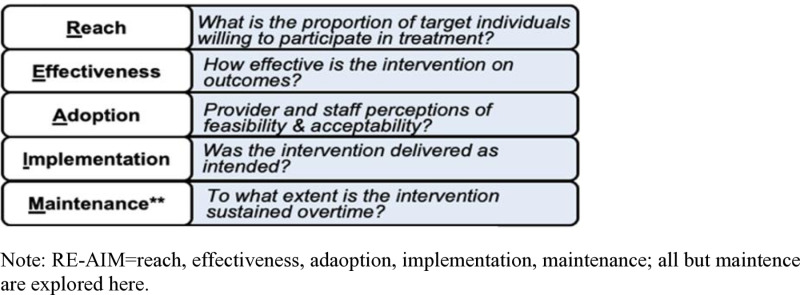
RE-AIM Model Indicators.

### Study setting and community-engaged approach

Research activities will take place in Eldoret, the fifth most populous city in Kenya, through collaboration with Moi Teaching and Referral Hospital (MTRH) and the Academic Model Providing Access to Healthcare (AMPATH), a consortium of North American and Kenyan institutions dedicated to healthcare delivery, research, and training. AMPATH administers a wide range of clinic- and community-based programs addressing both medical care and socio-economic empowerment, while MTRH houses Departments of Psychiatry and Psychological Counseling and operates an inpatient rehabilitation center. AMPATH also provides extensive research infrastructure and services that will support the implementation of this study. This collaboration builds on prior work between the investigators and AMPATH/MTRH, which demonstrated proof-of-concept for LEAD, showing high participant satisfaction and improvements in father depression, alcohol use, parenting, and child mental health outcomes.

Community engagement is central to the study design. This project follows principles of community-based participatory research (CBPR) [40]. In preliminary work, researchers collaborated with MTRH staff and local community leaders who identified a need for mental health services for fathers. To guide the ongoing research, a Community Advisory Board (CAB) has been established, comprising MTRH representatives, community leaders, researchers, and caregivers. The CAB will meet one to two times annually to provide input on study implementation, cultural and ethical considerations, dissemination, and future research priorities.

### Ethical approvals

The study has received IRB approval from Moi Teaching & Referral Hospital/Moi University College of Health Sciences-Institutional Research and Ethics Committee (MTRH/MU-IREC; FWA: 00003128). Florida International University (FIU) has executed a reliance agreement recognizing MTRH/MU-IREC as the IRB of record in accordance with U.S. regulations governing collaborative international research. This work aligns with Kenya’s Mental Health Policy 2015–2030 [34] and the World Health Organization’s Sustainable Development Goal 3.4, which seeks to reduce premature mortality from noncommunicable diseases and promote mental health and well-being.

### Timeline

Recruitment will begin April 2025 and remain ongoing likely until April 2026 or until 102 fathers are enrolled. Data collection is anticipated to be complete by October 2026 with results expected February 2027. At the time this manuscript was submitted (5/1/2025) recruitment had begun.

### Description of subject population

Fathers will be the primary subject population who will participate in the intervention and complete assessments. The other samples include female partners/co-Caregivers, youth, peer-father counselors, and implementation stakeholders; they will complete assessments only. Table 1 provides an overview of the subject populations and required target sample numbers.

**Table 1:** Subject Sample Sizes and Populations.

Subject Population	Number of completers required to accomplish study aims	Projected number to be enrolled to obtain required number of completers	Age range
Fathers	102	142	18-65
Female Caregivers/Partners	102	102	18-65
Youth	102	102	8- 17 years and 11 months
Peer Father Counselors	12	12	18-65
Implementation Stakeholders	14	14	18-65

### Eligibility criteria

Inclusion criteria are as follows: [1] male between the ages of 18 and 65 years; [2] currently living with and responsible for at least one child between the ages of 8 and 17 years; [3] screening positive for depressive symptoms, defined as a score greater than 5 on the Patient Health Questionnaire (PHQ-9); [4] any reported alcohol use within the past 45 days, indicated by a score of 1 or higher on the Alcohol Use Disorders Identification Test (AUDIT); [5] child at risk for mental health difficulties, defined as a score greater than 13 on the Strengths and Difficulties Questionnaire (SDQ) reported by any caregiver; and [6] willingness of the co-caregiver and the target child to participate in study assessments (consistent with previously piloted strategies).

Exclusion criteria are as follows: [1] severe depressive symptoms, defined as a PHQ-9 score greater than 19; [2] severe alcohol use disorder requiring medical management, defined as an AUDIT score of 20 or above; [3] history of violent legal offenses (screened with a single-item question); [4] indicators of severe interpersonal violence, assessed using key items from the Conflict Tactics Scale (CTS). If either the father or the co-caregiver endorses that the father “punched or hit my partner with something that could hurt” or “kicked my partner,” the dyad will be excluded. If either party endorses the father engaging in more severe violence (e.g., “used a knife or gun,” “choked,” “slammed against a wall,” “beat up,” or “burned/scalded on purpose”), they will also be excluded; [5] inability to provide informed consent or complete study procedures in Swahili or English; and [6] history or current presentation of serious mental illness or other drug dependence (‘yes’ response in two or more questions in CAGE- AID).

### Female partners/co-caregivers (sample 2)

Co-caregivers or female partners are individuals aged 18–65 years who are either the partners of eligible fathers (Sample 1) and/or help care for the fathers’ child (often the wife or mother of the child). Eligible co-caregivers must be willing to complete assessments related to family relationships and the child’s mental health. Inclusion and exclusion criteria for co-caregivers are detailed in Appendix Table A1.

### Youth (sample 3)

Sample 3 consists of children aged 8–17 years and 11 months who are the children of eligible fathers (Sample 1). Youth must provide assent and have caregiver permission to participate in assessments regarding family relationships and their own well-being. Inclusion and exclusion criteria for youth are detailed in Appendix Table A2.

One child will be selected per family to participate. The target child will be identified through a multi-step process: caregivers will first be asked to consider children within the eligible age range and identify the child they are most concerned about. If multiple children meet these criteria, preference will be given to the child they are most concerned about of the two, the child who is most likely to be home for later assessment (i.e., not at boarding school). Caregivers will then complete a screener (the Strengths and Difficulties Questionnaire, SDQ) for the identified child. In cases where caregivers disagree about which child is most affected, caregiver confidence in their responses (i.e., knowledge of the child) will be considered. A similar procedure was used successfully in the preceding proof-of-concept study. Youth do not need to be the biological child of the participating father but must be under his primary care (e.g., regular contact, financial or caregiving support).

### Peer-father counselors (sample 4)

Peer-father counselors are individuals aged 18–65 years who are identified and selected to deliver the LEAD intervention. Eligible individuals must attend training on the intervention. Inclusion and exclusion criteria for peer-father counselors are detailed in Appendix Table A3.

### Implementation stakeholders (sample 5)

Implementation stakeholders are individuals involved in supporting the delivery of the LEAD intervention who are not serving as peer-father counselors. This group is expected to include approximately four supervisors, five community leaders, and five hospital personnel. Inclusion and exclusion criteria for implementation stakeholders are detailed in Appendix Table A4. Unlike peer-father counselors, implementation stakeholders do not provide direct counseling but instead contribute to broader implementation activities. Stakeholders will be interviewed as part of Aim 3 to assess feasibility, acceptability, and implementation processes.

### Recruitment procedures

#### Fathers and their families [female partner/co-caregiver and youth].

Fathers and their families [female partner/co-caregiver and youth] will be approached by community leaders (including community health workers when relevant) accompanied by study staff. Community leaders are individuals who are well recognized and connected in the community through informal and formal structures such as chiefs or elders; very knowledgeable of community and gatekeepers to community members [27,35]. Community leaders will be accompanied by project staff to assess initial interest of fathers and conduct consenting and eligibility screening. As such recruitment will most often happen in private locations at people’s homes and in some cases, if preferred by the participant, a common community setting (e.g., school) or a MTRH office.

#### Peer-father counselors.

To recruit peer-father counselors (n = 12), we will employ previously used and tested strategies in the area [27,35] recruiting through community leaders as well. We will ask leaders to identify adult men (ages 18–65) who are fathers, seen as role models, show a willingness to learn and listen, are respected in the community. Leaders will nominate around candidates. With leaders, study staff will reach out to potential peer father candidates to explain the study. Candidates who are interested will be invited to an interview. During interviews, candidates will learn more about the study and be rated on natural counseling abilities, interest, availability, and willingness to learn by supervisors through questions and a role play; a subset will be chosen and invited to training. After a 10-day training, around 12 final peer-father counselors will be selected. They will be chosen based on (a) post- training clinical skills assessed during role plays with the Enhancing Assessment of Common Therapeutic (ENACT) Factors scale— a measure of clinical competencies for lay providers (> 2 preferred) [36]; (b) knowledge evaluated with written test (> 65% correct); and (c) ability to use manual, receptivity to feedback, and willingness to learn observed and tracked.

#### Implementation stakeholders.

Implementation stakeholders a who participate in the delivery of the LEAD study, who are not counselors, will be invited by the study staff to complete surveys and interviews conducted by a non-study member interviewer.

### Consenting and assenting procedures

Consent and assent procedures will be conducted prior to study participation, with forms provided in English or Swahili based on participant preference. Trained Kenyan staff psychologists will explain the study procedures, risks, voluntariness, confidentiality, and answer any questions.

#### Fathers, female partners/co-caregivers, and youth.

Consenting will occur sequentially: fathers first, then co-caregivers, and finally youth assent if caregiver permission is granted. Consent will be obtained in person using written forms and REDCap questionnaires. In line with common practice in global mental health research, consent will be obtained prior to eligibility screening to foster trust and transparency [27].

Father eligibility (Sample 1) is contingent on co-caregiver (Sample 2) participation, as caregivers report on family functioning and child mental health to confirm eligibility. Fathers will first complete consent and eligibility screening. Fathers excluded due to severe symptoms or reported violence will be referred to higher-level services at MTRH.

If the father is preliminarily eligible, consenters will request permission to invite a co-caregiver for screening. To maintain transparency and minimize risk, caregivers are informed that specific assessment information is private. This process also prioritizes female caregiver safety by ensuring voluntary participation and avoiding hidden study involvement.

Co-caregivers will be consented and screened in a private location. If privacy cannot be ensured, the session will be rescheduled. Co-caregivers will complete the Strengths and Difficulties Questionnaire (SDQ) and selected Conflict Tactics Scale (CTS) items [37,38]. If severe violence is indicated, staff will follow WHO ethical and safety guidelines. The local PI will be contacted if higher-level referral support is needed.

If both father and co-caregiver screenings confirm eligibility, assent from the target child (Sample 3) will be obtained. Child assent will be obtained in writing, using developmentally appropriate language.

#### Mental health and alcohol use referrals.

Individuals screened out due to severe symptoms will be referred to services at MTRH, including brief assessments, medication consultations, counseling, and/or psychiatric consultation, medication management, or violence support services. Referral protocols will follow WHO guidelines [39]. Youth scoring in the 20–40 SDQ range will also be referred to appropriate services, with referrals provided both to caregivers and youth aged 14 and older.

#### Peer-father counselors and implementation stakeholders.

Peer-father counselors and implementation stakeholders (Samples 4 and 5) will provide written consent using an information sheet administered by a trained research assistant.

### Study procedures

#### Data collection and assessment.

Assessments will be conducted at four timepoints: baseline, immediately post-LEAD, one-month post-intervention, and three months post-intervention (Fig 1). At baseline, all participants will receive referrals to care-as-usual services. Waitlist control (WL) participants will be assessed at each timepoint and offered LEAD following final assessment (~4.5 months later). (Given limited availability of outpatient services, this design ensures no care is withheld; participants with severe clinical concerns will be connected to existing referral pathways for urgent care).

At each assessment point, fathers, co-caregivers, and the designated child will complete evaluations of family functioning and mental health, following procedures piloted in our previous study [27]. Fathers randomized to LEAD will additionally complete proximal outcome measures during treatment sessions (e.g., activity completion) to evaluate short-term mechanisms of change (Table 2, Aims 1 and 2).

**Table 2:** Aim 1 and Aim 2 Outcomes.

Outcome	Construct	Measures	Reporter	Aim	Alpha in Kenya
*Primary*	Depression Symptoms	PHQ-9 [40]	F	1	0.91
*Secondary*	Alcohol Use; Gender Norms	AUDIT [41]; GEM [41]	F	1	0.89; 0.56
	Disrupted Parenting	APQ [42]	F, P, C	1	F = 0.78, P = 0.85, C = 0.92
	Interparental Problems	Togetherness Scale [43]	F, P, C	1	F = 0.95, P = 0.96, C = 0.95
	Child Mental Health	SDQ [37]	F, P, C	1	F = 0.75, P = 0.75, C = 0.75
*Proximal Mechanism*	Treatment Engagement	Attendance	Tracked	2	(% attended)
	Activity Completion	Homework Completion	LC, T	2	(% complete)
	Positive Reinforcement	Activity Emotion Valence	LC, T	2	(% + or –; yes/no)
Covariates	Age, Religion, Number of children				

*Note: F = father; P = partner/co-caregiver; C = child; LC = lay counselor; T = tracked; PHQ = Patient Health Questionnaire; AUDIT = Alcohol Use Disorder Identification Test; GEM = Gender-Equitable Men; APQ = Alabama Parenting Questionnaire; SDQ = Strengths and Difficulties Questionnaire*

**Table 3:** Aim 3 Assessment of Implementation Outcomes.

Measure	Reporter	RE-AIM Domain
***Acceptability***		
Semi-structured interview	F	*Adoption*
Focus Groups	PF, S, CL, H	
Acceptability of Intervention Measure (4-item Survey)	PF, S, CL, H, F	
Intervention Appropriateness Measures (4-item survey)	PF, S, CL, H, F	
***Feasibility***		
Father participation: % enrolled & % excluded	Tracked	*Reach*
Fidelity Checklist: *Adherence & Delivery Quality*	Coded	*Implementation*
Counselor Competency (ENACT Scale)	Coded	
Retention: Attendance & Attrition	Tracked	
Semi-structured interview	F	
Focus Groups	PF, S, CL, H	
Feasibility of Intervention Measure (4-itme survey) [44]	PF, S, CL, H, F	

PF = Peer-father counselor; S = Supervisor; F = Father; CL = community leaders; H = Hospital Staff; RA’s complete assessments; ***Effectiveness Aim 1, 2 focus***.

A sub-sample of LEAD-arm participants will participate in qualitative interviews one month after completing LEAD, including six fathers, six co-caregivers, and six children selected at random. Additional interviews and transcript analyses will be conducted with approximately 20 fathers exhibiting different patterns of response or non-response based on pre-post assessments. Peer-father counselors (N = 12) and delivery stakeholders (e.g., supervisors, community leaders, MTRH personnel) will complete post-intervention focus group discussions and brief surveys to evaluate implementation processes.

Assessments will be conducted by trained research assistants, blinded to participant condition, using secure tablets and REDCap data entry systems. An outside consultant will review incoming data for quality and accuracy. Assessments will be administered verbally unless participants request an alternative format. Project psychologists and psychiatrists will be on call for clinical support as needed. Data will be further monitored by a Data Safety and Monitoring Board who will review study data and progress at least once per year following recruitment.

#### Intervention.

**Fig 4:**
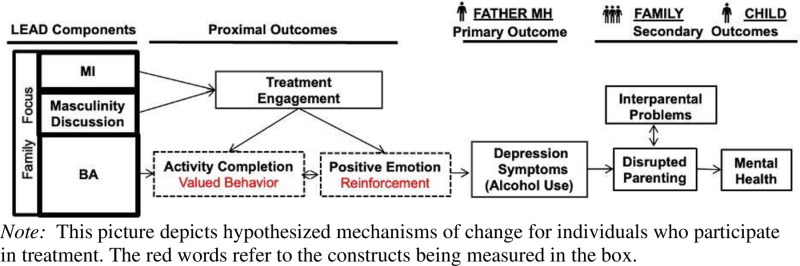
Hypothesized Pathways of Change.

Learn, Engage, Act, Dedicate (LEAD) is comprised of behavioral activation (BA), motivational interviewing (MI), and discussions of masculinity. LEAD has family focus throughout. The goal of LEAD is to target fathers’ depression symptoms and common comorbidities, such as drinking [42]. LEAD is guided by a manual (available in Swahili and English). It includes five, 60–90-minute weekly sessions. LEAD was adapted for context based on formative work. See Giusto et al., 2022 for in-depth development process. Fig 4 depicts hypothesized change pathways. Table 4 shows LEAD activities; each session begins with a mental health and alcohol use assessment and review of activity completion and ends with homework to monitor activities. Giusto et al. 2022 describes the intervention in depth. We briefly describe the three primary components of treatment here.

**Table 4:** Intervention Schedule.

LEAD INTERVENTION SCHEDULE	
**Session 1** **MI**	Pros and Cons of Change: Self & Family Importance Scaling Question Readiness Scaling Question Confirm Commitment Program Vision for Self & Family Value Selection: Self & Family Program Invitation Discuss Treatment Rationale Homework: Track Mood, Drinking. Notice Positive.
**Session 2** **BA**	Mental Health Assessment Homework Review: Connect mood to behavior Client’s Relationship with Father: Positive & Negative* Client’s Parents’ Relationship: Positive & Negative* Conceptions of Masculinity: Helpful & Hurtful* Value Selection: Self & Family Activity Scheduling Homework: Complete activities. Track drinking.
**Session 3** **BA**	Mental Health Assessment Homework Review: Connect mood to behavior Refusal Skills Value Selection: Self & Family Activity Scheduling Homework: Complete activities. Track mood.
**Session 4** **BA**	Mental Health Assessment Homework Review: Connect mood to behavior Refusal Skills Value Selection: Self & Family Activity Scheduling Homework: Complete activities. Track mood.
**Session 5** **BA**	Mental Health Assessment Homework Review: Connect mood to behavior Value Selection: Self & Family (Client-directed) Activity Scheduling (Client-directed) Staying on the Path: People and Strategies to Continue Change Lessons Learned: Treatment Review Graduate.

*Note: BA = Behavioral Activation; MI = Motivational Interviewing*

Brief BA has demonstrated efficacy in reducing depressive symptoms and comorbid alcohol and substance use across diverse contexts [24], including when delivered by lay counselors in LMICs [33,45]. LEAD incorporates core BA components, including activity monitoring, values assessment, activity scheduling, and skills training [22,46]. BA offers a structured, straightforward approach that targets maladaptive reinforcement patterns, addresses negative emotional triggers and behaviors linked to depression, and anchors interventions in participant values—making it both potent and conducive to cultural adaptation. The BA model posits that increasing engagement in pleasant and value-guided activities strengthens contact with positive reinforcement, thereby reducing depressive symptoms and promoting healthy behaviors. Improvements in drinking and depression occur by reinforcing healthy behaviors and addressing shared mechanisms of reinforcement [46].

Motivational Interviewing (MI), introduced in Session 1, has shown efficacy in increasing engagement in mental health treatment and reducing drinking behaviors, including when delivered by lay counselors [25]. MI enhances intrinsic motivation and reduces ambivalence about change [47]. LEAD integrates MI strategies throughout Sessions 1–5 to engage men in treatment, strengthen commitment to addressing personal goals, and build self-efficacy. For example, counselors use reflective strategies to reinforce successes during homework reviews [48].

Masculinity discussion strategies, introduced in Session 2, aim to broaden traditional conceptions of masculinity to include care, nurturance, and active family engagement. These discussions explore fathers’ beliefs about family roles, alleviating pressure associated with provider-only identities [49]. Such strategies have been successfully delivered by lay counselors in sub-Saharan Africa and have been shown to increase men’s engagement in family life [50].

#### Waitlist Control (WL).

The intervention will be compared to a waitlist control group. All participants regardless of assignment will be given referrals to care at baseline. Those randomized to WL will complete assessments and be monitored at each timepoint; they will be offered LEAD following the last assessment. In a pilot, a control allows for a realistic examination of recruitment, randomization, implementation of LEAD, assessment procedures, and retention. If safety concerns arise, referrals and safety procedures will be implemented.

#### Peer-counselor training & supervision (i.e., preparing to deliver).

***Training:*** A 10-day training, following piloted procedures, focuses on core clinical skills; specific session content; and safety planning. Training procedures are commensurate with other task-shifted (i.e., those that use peer or lay providers) interventions [51,52]. Trainees will be compensated for their time. Training will be conducted in Kiswahili directly or through live translation. ***Supervision:*** Supervisors for peer-father counselors will be local Kenyan individuals with bachelor’s degrees in psychology or in their final year of psychology training for their bachelors who are trained in LEAD. Local supervisors will consult with team leads. Supervision will use a tiered approach typically used in task- shifted LMIC intervention delivery [53].

### Sampling and power

Anticipating loss to follow up of ~10%, power was calculated conservatively based on participation of 90 fathers randomized 2:1 (LEAD = 60, WL = 30). We will explore clinical change between groups to inform future testing. Based on the distribution of change in PHQ-9 scores observed in our proof-of-concept study, I will have 80% power to detect a moderate 0.60 to large > 1.00 effect size of continuous outcomes using a two-sides z-test, alpha level of 0.05. The sample also allows exploratory structural equation modeling of pathways (n=~75; 5 variables*n = 15). Across analyses, we are not powered to definitively estimate effects.

### Data collection

Survey data will be collected on tablets that will be password protected and uploaded to a secure password protected server. No names will be collected on assessment materials (all deidentified). Sessions and qualitative interviews will be audio recorded on secure devices and deleted once they are uploaded to the secure drive. Written materials such as consent and assent forms will be stored in a locked drawer in a locked office.

### Data analysis by aims

#### Aim 1: Explore preliminary effectiveness of LEAD on father mental health.

Primary and secondary outcome measures are shown in Table 2 and 3. analysis will explore changes in depression and secondary outcomes from baseline to 3-month post-treatment based on intention-to-treat. Descriptive measures will be used to summarize data. Depression changes between baseline and post, 1-month post, and 3-month follow-up will be compared in the LEAD versus WL arms through mixed-effects linear regression models, with fixed effects for study condition, time, and the interaction between condition and time. We will include random effects to account for correlation of observations within individuals and individuals nested within peer-counselors. For secondary outcomes, we will take the same approach, evaluating measures by different respondents. We will conduct sensitivity analysis to see if change varies by respondents (e.g., child versus fathers).

#### Aim 2: Using mixed methods, explore the mechanisms driving change in father mental health, father parenting, and child mental health.

See Fig 3 for hypothesized pathways of change. It depicts LEAD driving healthy behavior and positive reinforcement/emotion [measured during LEAD], which will improve father depression [post-LEAD], which will improve parenting [1-month post] and improve child MH [3-month post].

Table 1 lists proximal outcomes linked to change in depression, as well as the secondary outcomes hypothesized to change child MH. Proximal outcomes – healthy activity completion and positive reinforcement – will be assessed through activity schedules completed as part of LEAD homework. Activity schedules will include the scheduled activities, whether the patient engaged in the activity, and emotion felt following engagement. These will be completed in session and checked by supervisors when reviewing session recordings for supervision. Emotions will be coded as ‘positive’ (e.g., pride) or ‘not positive’ (e.g., boring). This will result in three metrics: % of activities completed, frequency of healthy activity, and % of positive reinforcement associated with completed activities. Treatment engagement will be operationalized as attendance.

Qualitative data will be used to contextualize quantitative findings and generate new mechanistic hypotheses for a larger trial that considers social determinants of health. Data sources will be [1] *semi-structured interviews* with randomly selected fathers (n = 6), co-caregivers (n = 6), and children (n = 6) exploring perceived change/no change and reasons for change/lack of change, including the role of norms and poverty, 1 month after LEAD; and [2] *LEAD session transcripts* from men showing different change patterns (response/non-response) based on pre-post scores (n = 20); patterns include (a) no change in depression or secondary outcomes (n = 5), (b) depression change, no secondary outcome change (n = 5), (c) depression change, secondary outcome change, (d) no depression change, secondary outcome change (n = 5).

#### Mixed-methods analysis.

The study will use an *equal-status, convergent parallel mixed-method design*, meaning both the quantitative and qualitative findings will be equally valued, analyzed separately, then integrated guided by hypothesized change pathways. ***Quantitative:*** I will explore pathways using structural equation modeling with data from Baseline, During Treatment [proximal mechanisms], Post, 1-month, and 3-month post. We will conduct a path analysis given we are not using latent variables. We will explore 3 pathways: [1] the indirect effect of LEAD on father depression via increased behavior and positive emotion, [2] the direct and indirect effect of father depression on child MH via parental interactions, and [3] if we detect relationships in path 1 and 2, we will explore the direct and indirect effects of LEAD on child mental health via father’s depression. If no relationships are found, we will explore other potential drivers of father depression (path 1), such as baseline severity, that may influence change. ***Qualitative:*** We will employ the Framework Method [54] with inductive and deductive coding. Two RAs will familiarize themselves with interview and session transcripts. Next, they will inductively open-code transcripts line-by-line, then deductively code transcripts guided in part by hypothesized pathways (Fig 3). The resulting codes will be operationalized into a final analytic framework and codebook [54]. To establish reliability, a Project Coordinator (PC) and study staff will independently code 10% of transcripts. We will review definitions, codes, and resolve differences until interrater reliability (Kappa) is higher than 0.70. Any additional codes emerging will be added to the codebook. Once data are coded, they will be charted and assessed with a matrix. Results will elucidate and contextualize mechanisms, and potential moderators, to test in a future trial.

#### Aim 3: Explore the feasibility and acceptability of implementing LEAD.

Guided by RE-AIM outcomes, we will assess indicators of implementation success using a process evaluation of LEAD’s “*acceptability*” and “*feasibility*”. This will include a qualitative exploration of social determinants, such as norms, economic hardship, and financial stress, as barriers and facilitators of engagement and retention. Table 3 highlights these measures. Acceptability is defined as satisfaction with LEAD and its implementation, and willingness to initiate the program for the population. Feasibility is defined as the extent to which LEAD engages and retains fathers and can be implemented as intended. This consists of [1] Patient *participation*; [2] Peer-father *fidelity* to LEAD measured with a previously developed and piloted checklist assessing step adherence (Yes/No) and quality of step delivery from poor [4] to excellent [1]; [3] *general counselor competency* measured with 8 items from the ENhancing Assessment of Common Therapeutic Factors scale developed for evaluating lay counselor-delivered treatment in low- resource settings [36] (also previously piloted with LEAD [27]; [4] *Retention* (for both arms; details below); and [5] *Perceptions of feasibility, acceptability, and barriers/facilitators* to implementation. Perceptions will be explored in focus groups with the 12 peer-father counselors, 4 supervisors, 5–10 community leaders who recruited the counselors, and 5–10 MTRH staff; in the focus groups, a brief survey measure of feasibility will also be administered [44]. Father perceptions of feasibility will also be assessed during interviews conducted during Aim 2.

#### Analysis.

Interviews and focus groups will be analyzed using methods described in Aim 2. Deductive codes will be guided by RE-AIM domains and acceptability/feasibility constructs described above (e.g., barriers/facilitators). Participation will be analyzed as % of eligible fathers who enroll in LEAD and % excluded; *retention* will be % of fathers attending ≥ 1 session, attending ≥ 75% of sessions, and dropping out. We will explore retention rates across both arms. To examine fidelity and counselor competency, all sessions will be audio recorded; 25% will be transcribed, translated to English, and reviewed by the PC. We will conduct consensus ratings on the first 4 transcripts, then reach 80% agreement on ratings before independently rating the remainder (% agreement was chosen to be directly interpretable [55].) Fidelity will yield two scores: [1] % of steps completed based on proportion of LEAD components delivered as intended and [2] mean quality scores across steps for each case. Counselor competency will be calculated as score averages per counselor session. Results will be discussed with the CAB to identify and refine implementation strategies for a fully powered Hybrid trial for a future R01 proposal in an R01 in which RE-AIM will guide continued evaluation of implementation outcomes, including examination of Maintenance (*Outcomes:* Cost, Sustainability) and a more in-depth examination and test of Reach (*Outcome:* Penetration).

## Discussion

This protocol describes a pilot hybrid type 1 study using a randomized waitlist-control design to evaluate the preliminary effectiveness of *Learn, Engage, Act, Dedicate* (LEAD), a peer-delivered intervention for fathers targeting depression and alcohol use, and its impact on family and child mental health outcomes. Fathers will be randomized 2:1 to LEAD or a waitlist control. In addition to assessing preliminary clinical outcomes, the study will explore implementation outcomes, including feasibility, acceptability, reach, and fidelity. Findings will inform the design of a future hypothesis-testing hybrid trial to evaluate both LEAD effectiveness and implementation strategies.

### Implications and significance

Few mental health interventions explicitly target fathers or address social determinants such as gender norms in both treatment content and delivery. LEAD aims to engage men through a healthy masculinity lens, working with peer-fathers as lay counselors to facilitate discussions that expand traditional conceptions of masculinity beyond financial provision to include care and nurturance. By further exploring barriers and facilitators to men’s engagement and retention in care, the study can refine delivery strategies critical to improving men’s mental health service utilization.

This trial also directly addresses a major gap in the literature linking father depression and alcohol use to child mental health outcomes. Research has historically focused on maternal depression, despite growing evidence that paternal mental health substantially influences child well-being [8,56]. Targeting fathers may provide a parsimonious strategy to improve multiple, interconnected outcomes — father mental health, family functioning, and child mental health — and may contribute to breaking the intergenerational transmission of mental health risk. In addition to assessing whether father treatment impacts child outcomes, the study will explore mechanisms of change to identify key targets for future interventions.

The study’s design prioritizes scalability through a brief, task-shifted intervention format. LEAD integrates two evidence-based approaches — behavioral activation and motivational interviewing — to address depression, alcohol use, and family relationship problems concurrently. Despite the common co-occurrence of these issues among men [57,58], many interventions fail to address them holistically, limiting scalability. LEAD’s focus on training peer-fathers, rather than traditionally trained lay providers who are often women, promotes affordability, cultural relevance, capacity building, and sustainability for long-term implementation.

### Potential challenges

As with any study, challenges are anticipated. Although not all can be predicted, several potential issues were raised by the study team and Community Advisory Board. One concern is that eligibility criteria may be too restrictive, potentially limiting recruitment. While strict criteria support internal validity, we will monitor recruitment closely and adjust if criteria significantly impact external validity.

A second potential challenge relates to father engagement and retention, a broader issue the study seeks to better understand. Successfully recruiting and maintaining father participation remains a key priority. Finally, balancing partnership requirements across U.S. and Kenyan institutions is an ongoing challenge in global mental health research. Although these processes can be time-intensive, the study team has extensive experience navigating cross-institutional collaborations and is committed to fostering equitable, sustained partnerships.

## Conclusion

This protocol describes a pilot hybrid type 1 study using a randomized waitlist-control design to evaluate the preliminary effectiveness and implementation outcomes of LEAD***.*** By targeting father depression and alcohol use — two critical but often overlooked drivers of family and child mental health problems — through a task-shifted model, this study addresses significant gaps in the global mental health literature, which has historically prioritized maternal mental health and underexamined paternal pathways of risk and resilience. If successful, this work can contribute to advancing family-centered approaches to mental health intervention, offering a model for engaging men in low-resource settings and informing broader strategies for sustainable, community-anchored mental health care.

## Supporting information

S1 File SPIRIT Checklist. (DOC)

S2 File Appendix Protocol Paper. (DOCX)

S3 File IREC Protocol. (PDF)
