# Supplementary material for: Protocol for a Hybrid-type 1 pilot study of a randomized control trial of a brief, peer-delivered treatment to improve father depression and child mental health in Kenya
Source: PLoS One. 2025 Jun 26;20(6):e0325902. doi: 10.1371/journal.pone.0325902 (PMC12200834; doi:10.1371/journal.pone.0325902)
Supplement: S2 File — (DOCX) [file pone.0325902.s002.docx]

Appendix

**Table A1. Female Caregivers/Co-Caregivers Inclusion/ Exclusion Criteria**

| CRITERION | METHOD OF ASCERTAINMENT |
| --- | --- |
| **Inclusion:** |  |
| Partner or co-caregiver of eligible father | Demographic Question |
| 18 to 65 years of age | Demographic Question |
| Willingness to participate | Demographic Question/Consent |
| **Exclusion:** |  |
| Inability to provide informed consent, and/or complete procedures in Swahili or English (orally). | Consenting Process |

**Table A2. Youth Inclusion/ Exclusion Criteria**

| CRITERION | METHOD OF ASCERTAINMENT |
| --- | --- |
| **Inclusion:** |  |
| Child of eligible father | Demographic Question (not necessarily biological but male caregiver is responsible for the care of the child and  involved in the child’s life.) |
| 8 to 17 years 11 months of age | Demographic Question |
| Willingness to participate | Demographic Question/Assent |
| At risk of mental health problems | Either caregiver reports a score of > 13 to 40 on the Strengths and Difficulties Questionnaire (SDQ); [Score Range 13-40]. |
| **Exclusion:** |  |
| Inability to provide informed assent, and/or complete procedures in Swahili or English (orally). | Assenting Process |
| Not at risk for MH problems | Score <13 to 40 on the SDQ as reported by both caregivers |

**Table A3. Peer-Father Counselor Inclusion/Exclusion Criteria**

| CRITERION | METHOD OF ASCERTAINMENT |
| --- | --- |
| **Inclusion:** |  |
| Deliver or potentially deliver LEAD | Individuals identified and chosen to potentially deliver LEAD (nominated by leaders) who attend training |
| 18 to 65 years of age | Demographic Question |
| Willingness to participate | Demographic Question/Consent |
| **Exclusion:** |  |
| Inability to provide informed consent, and/or complete procedures in Swahili or English (orally). | Consenting Process |

**Table A4. Implementation Stakeholder Inclusion/Exclusion Criteria**

| CRITERION | METHOD OF ASCERTAINMENT |
| --- | --- |
| **Inclusion** |  |
| Involved in the delivery of LEAD | Project Roles: Supervisor, Community Leaders, and Hospital Staff who worked on or helped deliver LEAD |
| 18 to 65 years of age | Demographic Question |
| Willingness to participate | Demographic Question/Consent |
| **Exclusion:** |  |
| Inability to provide informed consent, and/or complete procedures in Swahili or English (orally). | Consenting Process |

**Table A5. Schedule of assessments**

| **Construct** | **Measure** | **Repo​​rter** | **Timepoint** |
| --- | --- | --- | --- |
| Depression Symptoms | Patient Health Questionnaire-9 | F | BL  IP  1MP  3MP |
| Drinking | Alcohol Use Disorder Identification Test | F | BL  IP  1MP  3MP |
| Gender Norms | Gender-Equitable Men’s Scale | F | BL  IP  1MP  3MP |
| Disrupted Parenting | Alabama Parenting Questionnaire (adapted) | F  Co  C | BL  IP  1MP  3MP |
| Interparental Problems | Family Togetherness Scale | F  Co  C | BL  IP  1MP  3MP |
| Child Mental Health | Strengths and Difficulties Questionnaire | F  Co  C | BL  IP  1MP  3MP |
| Treatment Engagement | Attendance | Tracked | During Treatment |
| Activity Completion | Homework Completion | PF, T | During Treatment |
| Positive Reinforcement | Activity Emotion Valence | PF, T | During Treatment |
| Acceptability | Semi-Structured Interview | F | Post-LEAD |
|  | Focus Groups | PF, S, CL, H | Post-LEAD |
|  | Acceptability of Intervention Measure (4-item Survey) | PF, S, CL, H, F | Post-LEAD |
|  | Intervention Appropriateness Measures (4-item Survey) | PF, S, CL, H, F | Post-LEAD |
| Feasibility | Father participation: % enrolled & % excluded | Tracked | During Treatment |
|  | Fidelity Checklist; Adherence & Delivery Quality | Coded | During Treatment |
|  | Counselor Quality (ENACT Scale) | Coded | During Treatment |
|  | Retention: Attendance & Attrition | Tracked | During Treatment |
|  | Semi-Structured Interview | F | During Treatment |
|  | Focus Groups | PF, S, CL, H | During Treatment |
|  | Feasibility of Intervention Measure (4-item Survey) | PF, S, CL, H | Post-LEAD |

Note:  F=Father; Co=co-caregiver; C=child; PF=peer-father counselor, T=tracked; BL=baseline; IP=immediately post-intervention; 1MP= 1-month post; 3MP=3-month post; *= Each measure transculturally translated, adapted, and tested in Kenya; S=Supervisor; F=Father; CL=Community Leader; H=Hospital personnel; RA’s complete assessments.
