# Supplementary material for: Protocol for a Hybrid-type 1 pilot study of a randomized control trial of a brief, peer-delivered treatment to improve father depression and child mental health in Kenya
Source: PLoS One. 2025 Jun 26;20(6):e0325902. doi: 10.1371/journal.pone.0325902 (PMC12200834; doi:10.1371/journal.pone.0325902)
Supplement: S3 File — (PDF) [file pone.0325902.s003.pdf]

A brief, task shifted treatment to improve father depression and child outcomes in Kenya: A pilot effectiveness implementation trial

Investigators:

Dr. Ali Giusto (Principal Investigator)

Dr. Florence Jaguga (MBChB, MMed Psych) (Co-investigator/ Advisor)

Declarations

Ali Giusto (PhD)
Florida International University, Department of Psychology

Signature...Ali Giusto...... Date 10.12.24

Florence Jaguga (MMed Psych)
Department of Mental Health, Moi Teaching & Referral Hospital

Signature ...[illegible]... Date 22nd October 2024

Contents

Declarations... 2
Abstract.. 4
Background.. 4
Specific Aims.. 6
Methods 7
Study design 7
Study setting.. 7
Recruitment & Inclusion Exclusion Criteria:7
Data Collection and Sampling and Power 11
Data Management and Analysis............ 12
TIMEFRAME 13
Ethics Consideration.............................. 14
Risks 14
Benefits 15
Informed Consent 15
Confidentiality 16
Ethical approval 17
Study Implications................................. 17
APPENDIX I: CONSENT/ ASSENT FORMS 18
APPENDIX II: Examples of Questionnaires/Surveys 29
Reference 35

Abstract:

Globally, depression is the leading cause of disability world worldwide, yet over 75% of people in low and middle-income countries (LMIC) do not receive treatment. This gap is especially large for men. Treating men is essential to reducing the global burden of depression given its impact on families and children with men's mental health (MH) predicting poor parenting and child MH. Although treating parent depression has been shown to improve parental MH, parenting, and child MH; men and fathers have been missing MH care and clinical trials. MH care relevant to fathers that address common barriers to MH, such as masculine norms and economic factors, are needed. This project seeks to conduct a pilot randomized control trial (RCT) using an effectiveness-implementation design in Kenya of a task-shared depression and alcohol use intervention for fathers. The project will build on preliminary work with AMPATH/MTRH that showed proof-of-concept for 'Learn, Act, Engage, Dedicate' (LEAD), a 5-session task-shifted behavioral activation intervention with motivational interviewing and masculinity discussion strategies for fathers in Eldoret that support pursuit of a pilot RCT (n=102 fathers). Specifically, we propose a pilot RCT with fathers randomized to either LEAD or a waitlist control group (with all referred to care as usual at enrollment) to (1) explore change in fathers' MH; (2) explore drivers of change in father MH, father parenting, and child MH (or non-response); and (3) explore the feasibility and acceptability of implementation.

Background:

Depression is the leading cause of disability worldwide[1], yet over 75% of people in low and middle-income countries (LMICs) do not receive treatment[2]. This gap is especially large for men[3], who initiate and stay in treatment at rates 4 times lower than women[4,5]. Improving treatment for men is essential to reducing the global burden of depression given its impact not only on men's wellbeing but also on their families' functioning and children's mental health

(MH)[6]. Men's MH impacts child MH both directly[7,8] and through interparental problems and impaired parenting[6,9,10]. Meta-analyses show that treating parental depression improves parenting and child MH[11]. Any comprehensive strategy to reduce the burden of child MH problems (the leading cause of disability among youth[12]) must consider fathers' MH[3]. Despite the impact of fathers' MH on families, fathers are missing from MH treatment[2,3]. Stigma[13], masculine norms[13], and drinking behavior[14] are barriers to men's MH care engagement and retention, worsen men's MH problems,[15] and worsen the consequence men's MH on families and children[16]. Consistent with the Family Stress Model,[17] formative qualitative work we led in Kenya showed a similar pattern: economic pressure and inability to provide were tied to men's depression symptoms, leading to drinking, arguments at home, and poor child MH[18]. Strategies to engage and treat men with depression must address these factors[19].

To address the lack of relevant services for fathers' depression, I, with a team of Kenyan and US clinicians and researchers, designed and collected preliminary data on LEAD (Learn, Engage, Act, Dedicate), a 5-session behavioral activation (BA) intervention delivered by peer-father counselors[18] that incorporates motivational interviewing (MI) and masculinity discussion strategies. BA, an efficacious intervention for depression[20], targets depression symptoms by replacing maladaptive behaviors (e.g., isolating, drinking) with valued behaviors[18,21]. MI, an efficacious treatment for drinking and treatment engagement[22,23], is used to engage men in LEAD and address drinking; masculinity discussions expand ideas of what it means to be a father beyond provider only. As a first step[24]**,** we conducted a proof-of-concept test of LEAD with 9 fathers and 3 peer-father lay counselors in Kenya using a multiple baseline single-case series design. LEAD was acceptable; fathers reported improvements on their depression and drinking, and co-caregivers/partners and children reported improvements in couple's problems, parenting, and child MH. No adverse events were reported[25].

Here we propose a pilot RCT[24,26] of LEAD using a Hybrid Type I effectiveness-implementation pilot design that will allow exploration of clinical *and* implementation outcomes simultaneously to inform a future fully powered RCT examining both LEAD effectiveness and implementation sustainability. Guided by the RE-AIM framework— an implementation framework to guide clinical and implementation evaluation[27], in this pilot

research, we will explore the preliminary effectiveness of LEAD (i.e., change in father MH; potential mechanisms of change in father and child MH) and its implementation (i.e., feasibility and acceptability), randomizing fathers (n=102[28]) with depression (2:1) to LEAD delivered by peer-father counselors or to waitlist control (offering LEAD after final assessment). All groups will be referred to care as usual at enrollment. I will explore clinical change between groups to inform a future definitive RCT recognizing that we are not powered to estimate definitive effectiveness of LEAD here.

Specific Aims

Aim 1. Explore the preliminary effectiveness of LEAD on father MH. The *primary outcome* is father depressive symptoms assessed by the Patient Health Questionnaire, previously validated in Kenya. *Secondary outcomes* include fathers' drinking, father parenting, interparental problems, and child MH (ages 8-17) reported by the father, co-caregiver, and one child immediately and 1-month and 3-months post-LEAD.

Aim 2. Use mixed methods to explore mechanisms driving change in father MH, father parenting, and child MH. I will use structural equation modeling to explore clinical mediators between LEAD and father and child MH: LEAD→ positive activities→ father MH→parenting→child MH. To explore patterns of response/non-response, I will conduct qualitative interviews (fathers, co-caregivers, children, peer-counselors) on perceived change pathways and analyze LEAD-session transcripts of families showing different change outcomes (e.g., all changed, none, father MH only, child MH only). Qualitative data will be analyzed using the Framework Method[29]. Data will be integrated to generate hypotheses and refinements.

Aim 3. Explore feasibility and acceptability of implementing task-shifted MH treatment for fathers. A mixed-methods process evaluation with fathers, peer-fathers, and delivery stakeholders will explore *implementation outcomes:* reach; retention; fidelity; feasibility; acceptability; and barriers to/facilitators of implementation, scale-up, and sustainability, including questions related to social determinants of health, e.g., gender norms, poverty, (interviews, focus groups, surveys).

Methods:

Study design:

This will be a pilot RCT using a hybrid type 1 implementation. Results will be evaluated using mixed methods (qualitative and quantitative methods).

Study setting:

Research will be conducted in Eldoret, the fifth most populated city in Kenya, in collaboration with MTRH and AMPATH. AMPATH administers clinic- and community-based programs for medical care and broader programs focused on poverty reduction and child wellness. MTRH has Departments of Psychiatry and Psychological Counseling and an inpatient rehabilitation center. AMPATH supports numerous research programs and services. Participants and peer providers will be recruited from the community (Eldoret and surrounding areas) as described below.

Recruitment & Inclusion Exclusion Criteria:

We will work with **community leaders, peer-leaders supporting community substance use groups, and** existing Community Health Workers (CHWs) to recruit and identify eligible fathers with depression, any drinking, and a child at risk for MH problems (n=102 **Table 1**), one target child for each father and a co-caregiver (partner or relative who can report on family and child). *We will recruit to achieve balance in child age (50% 8-12, 50% 13-17)*. All eligibility cut-off scores are based on validated measures. Inclusion includes the following: (1) Male between the ages of 18-65; (2) live with and be responsible for at least one child between the ages of 8 and 17 years of age; (3) screen positive for depression symptoms, operationalized as a score above 5 on the patient health questionnaire (PHQ-9); (4) Any reported alcohol use in the past 45 days measures with the AUDIT (score 1 or above); (5) child at risk of mental health issues as indicated by a score above 13 on the Strengths and Difficulties Questionnaire (SDQ) reported on by any caregiver; (6) willingness for co-caregiver and target child to participate in assessments (previously piloted strategy). Exclusion criteria includes: (1) Severe depression symptoms indicated a score above 19 on the PHQ-9; (2) Severe risk/likely alcohol dependence that warrants medical management indicated as a score 20 or above on the alcohol use disorder identification test (AUDIT); (3) Severe risk/likely substance use dependence that

warrants medical management indicated as a ‘yes’ response in two or more questions in CAGE-AID; CAGE-AID is a standardized tool which is an adaption of the CAGE used for conjointly screening alcohol and drug dependence (Maina, Elizabeth & Wagoro, Miriam & Angeline, Kirui & Lincoln, Khasakhala.,2017). The CAGE-AID, as a screener, has shown validity as a screening tool for both alcohol dependence and drug dependence (Brown, R. L., & Rounds, L. A.,1995). The screener was originally adapted from the CAGE a standardized tool for measuring alcohol dependence whose acronym stands for ‘Cut down, Annoyed, Guilty, Eye opener’ (4) violent legal offenses (one question); (5) Indicators of severe violence at home assessed with key items from the Conflict Tactics Scale (CTS); (6) Inability to provide informed consent of complete procedures in Swahili or English; (7) serious mental illness (current or history); We use a low depression threshold for inclusion, given men often underreport symptoms[30]; at this piloting stage, we will try to recruit men who have a child at risk for MH problems to explore how father treatment may influence child MH change (Aim 2). The youth does not have to be father’s biological child, just responsible for the care of the child and involved in the child’s life. The SDQ has shown sensitivity to change in Kenya[31,32].

We will work with the on-site PI, study staff, and the MTRH community-based substance use group lead to identify community and peer leaders including CHWs. [*Community leaders* can consist of chiefs, village elders, religious leaders, or individuals well-connected in communities; individuals with formal and informal community positions, longstanding community knowledge, and can represent diverse sectors of the community. *Peer-leaders* are individuals with lived-experience who lead community-based substance use groups within Eldoret and surrounding communities.] We will approach leaders about the study to assess their interest in helping recruit fathers and peer-father counselors. If so, we will describe the type of fathers we hope to recruit using lay-terms based on inclusion and exclusion criteria. Leaders will than work with the trained consenters, Kenyan RA(s) and/or the PC who are all Kenyan psychologists, to reach out to potential participants, assess interest, and conduct eligibility screening and consent and assent procedures. Consenters will be trained to provide

**Table 1. Father Recruitment Criteria**
**Inclusion Criteria**
Live with and be responsible for the care of at least one child aged 8-17 years
18 to 65 years of age
Elevated depression symptoms (PHQ-9 > 5)[33]<br>Any alcohol use (AUDIT>0)
Child at risk of MH problems (SDQ > 13)[34]
Willingness for family member to participate
**Exclusion Criteria**
Severe depression symptoms (PHQ-9 > 19)
Severe risk/likely alcohol dependence that warrants medical management (AUDIT > 20)[35]<br>Severe risk/likely substance use dependence that warrants medical management (‘yes’ score in two or more questions in CAGE-AID)
Violent legal offences
Any indicator of severe violence (CTS items)
Inability to provide informed consent, and/or complete procedures in Swahili or English (orally).

referrals to care in the area to all individuals who report problems or referrals to higher levels of care to those who need it. Referral will continue to include the community substance use groups run by MTRH when appropriate. Consenters will complete the consent process and conduct the eligibility screening first with the father and then with other family members when the father is eligible. Eligibility and screening will occur first with fathers, then co-caregivers, then the target child. The target child will be chosen based on age first, then highest SDQ score based on caregiver screeners. *All individuals who report symptoms will be referred to care as usual.*

Consenting and Safety Considerations: The study will follow WHO guidelines on conducting research with individuals who may be at risk of violence as a precaution. Guidelines will inform consent forms, procedures, and personnel training[36]. Procedures are detailed in Human Subjects 3.1. Briefly, with fathers, **trained consenters** (i.e., the study RA and PC and or other trained medical psychologists) will assess interest, complete consent, and conduct eligibility screening. Study inclusion/exclusion will be explained in general terms to not place any youth or co-caregivers at risk later if a family is ineligible. Scripts will be developed with our community partners. Fathers excluded due to severe symptomology or severe violence will be referred to a higher level of care within MTRH (Human Subjects 3.1). The CHW will ask eligible fathers for permission to invite a co-caregiver to answer screening questions about the child, emphasizing that all information beyond basic study detail will be kept private from the co-caregiver and vice versa (a process used in the proof-of-concept pilot). The co-caregiver will then be consented and screened in a private location (SDQ; CTS); if the CTS indicates presence or likelihood of severe violence, a safety assessment will be conducted and referrals provided while respecting women's autonomy[36,37]. Fathers' ineligible in this scenario will be revisited later by the CHW, asked dummy questions, and told they are ineligible using non-descript language; referrals and an appointment to care will be provided to MTRH. If the father is eligible after both screenings, the target child will complete assent and a brief screen (SDQ). ***Recruitment feasibility:*** Based on ongoing studies, we estimate recruiting 2-4 fathers per week. This rate will allow us to recruit 102 families within 24 months.

Peer-father counselor recruitment, training, supervision. To recruit, select, supervise, and train peer-father counselors, we will use approaches employed in our preliminary pilot that

demonstrated feasibility[25] . ***Recruitment and selection:*** We will recruit 12 peer-fathers through community leaders (e.g., religious leaders, chiefs, elders). We will ask community leaders to identify around 30 men (ages 18-65) who are fathers, are seen as role models, and show a desire to learn. We will then reach out to these men, explain the program and training, and if they are interested, invite them to be interviewed. We will interview the interested men about previous formal counseling experiences, availability and interest in learning counseling and participating on a project, and ask them to complete a brief counseling role play. After interviewing interested men, around 20 men will be invited to a training. Having about 20 men at training will provide a large enough pool from which to choose 12 final counselors. This is based on previous pilot rates of interview to final counselor selection. After training, of the 20, 12 will be selected based on their performance in training measured with the ENACT Scale, a scale assessing core counseling competencies previously used in Eldoret; supervisors' assessment of their willingness to learn new material and openness to feedback; and knowledge of the intervention assessed with a written examine. ***Training:*** A 10-day training spread over weekends and afternoons, following piloted procedures[25], will focus on core clinical skills; specific session content; and emergency and safety planning. Training procedures are commensurate with other task-shifted LMIC interventions[38,39]. Trainees will be compensated for their time. Supervisors, Dr. Jaguga, and I will lead training. Training will be conducted in Kiswahili directly or through live translation. ***Supervision:*** Supervisors for peer-father counselors will be four local Kenyan individuals with bachelors' degrees in Psychology or in their final year of psychology training for their bachelors who are trained in LEAD. Local supervisors will consult weekly with Dr. Jaguga and myself. Supervision will use a tiered approach often used in task-shifted LMIC intervention delivery[40]: 1) After a session, the supervisor debriefs with the counselor on immediate concerns (phone); 2) after listening to counselor sessions, supervisors consult with Dr. Jaguga and myself to solidify a supervision plan; 3) supervisors conduct supervision with counselors to plan the next session and practice skills.

Treatment Conditions

***Intervention*.** LEAD comprises behavioral activation (BA) and motivational interviewing (MI), as well as discussions of masculinity and a family focus throughout to target father's depression symptoms and

common comorbidities, such as drinking. LEAD is guided by a manual (in Swahili and English). It includes five, 60-90-minute weekly sessions. LEAD was adapted for context based on formative work. Each session begins with MH assessment and review of activity completion and ends with homework to monitor

activities. ***Brief BA*** has shown efficacy reducing depression symptoms as well as co-morbid alcohol and substance use across contexts[41–43] and when delivered by lay-counselors[44,45]. LEAD uses core BA components. ***MI*** (Session 1) has shown efficacy for increasing MH treatment engagement and reducing drinking behaviors, with strong effects when delivered by lay counselors[39,46,47]. LEAD uses MI strategies to engage men in the treatment and increase commitment to addressing problems. MI strategies are then integrated throughout BA[48] (Session 1-5) to enhance father motivation to complete activities as well as to build self-efficacy (e.g., when reviewing homework, counselors reflect successes).

Waitlist Control (WL)**.** Those randomized to WL will complete assessments and be monitored at each timepoint; they will be offered LEAD following the last assessment. In a pilot, a control allows for a realistic examination of recruitment, randomization, implementation of LEAD, assessment procedures, and retention. If safety concerns arise, referrals and safety procedures will be implemented.

Data Collection and Sampling and Power

We will compare LEAD to a monitored waitlist control (WL) on preliminary clinical and implementation outcomes. Using a random number generator in RStudio, we will randomize participants 2:1 to LEAD:WL, stratified by levels of drinking to ensure group exchangeability. Assessments will occur at baseline, immediately following LEAD, 1-month, and 3-month post-intervention. WL participants will be assessed at each timepoint; after the last assessment (~4.5 months), they will be offered LEAD.

Each father and his co-caregiver and designated child will complete assessments on family problems and MH at each timepoint (both arms); a procedure previously piloted[25]. For fathers in LEAD, proximal mechanism outcomes will be assessed during LEAD (e.g., activity completion; Table 2). A sub-set of fathers, co-caregivers, and children in the LEAD arm will complete qualitative interviews 1 month after LEAD; peer-father counselors (N=12) and delivery stakeholders (supervisors, community leaders, MTRH personnel) will complete focus group discussions and brief surveys 1-month post-LEAD. Trained Kenyan RAs will conduct assessments in

Table 2. Aim 1 and Aim 2 Quantitative Measures

Outcome	Construct	Measures*	Reporter	Aim	Alpha in Kenya
Primary	Depression Symptoms	PHQ-9[33]	F	1	.91
Secondary	Drinking; Gender Norms	AUDIT[35]; GEM[49]	F	1	.89; .56
	Disrupted Parenting$^+$	APQ[50,51]	F, Co, C	1	F=.78, P=.85, C=.92
	Interparental Problems$^+$	Togetherness Scale[52]	F, Co, C	1	F=.95, P=.96, C=.95
	Child Mental Health	SDQ[31,34]	F, Co, C	1	F=.75, P=.75, C=.75
Proximal Mechanism	Treatment Engagement	Attendance	Tracked	2	(% attended)
	Activity Completion$^+$	Homework Completion	PF, T	2	(% complete)

Swahili (with translation to English as needed) on secure tablets

	Positive Reinforcement[+]	Activity Emotion Valence	PF, T	2	(% + or –; yes/no)
Covariates	Age, Religion, Number of children				

***Notes**:* F=father; Co=co-caregiver; C=child; PF=peer-father counselor; T=tracked; PHQ=Patient Health Questionnaire; AUDIT=Alcohol Use Disorder Identification Test; GEM=Gender-Equitable Men; APQ=Alabama Parenting Questionnaire; SDQ=Strengths & Difficulties Questionnaire; *= Each measure transculturally translated, adapted, and tested in Kenya. += As per Figure 5, path 1 and 2 hypothesized mechanisms

with on-call access to a project psychologist/psychiatrist. Assessors will be blinded to condition. Assessments will be conducted verbally unless a participant prefers otherwise. Participants will be compensated for travel and time.

Power. Anticipating loss to follow up of ~10% (seen in similar trials[44]), power was calculated conservatively based on participation of 90 fathers randomized 2:1 (LEAD=60, WL=30). We will explore clinical change between groups to inform future testing. Based on the distribution of change in PHQ-9 scores observed in our proof-of-concept study, I will have 80% power to detect a moderate 0.60 to large > 1.00 effect size of continuous outcomes using a two-sides z-test, alpha level of 0.05. The sample also allows exploratory structural equation modeling of pathways (n=~75; 5 variables*n=15). Across analyses, we are not powered to definitively estimate effects.

Data Management and Analysis

Data will be collected on tablet that will be password protected and uploaded to a secure password protected server. No names will be collected on assessment materials (all deidentified). Sessions will be audio recorded on secure devices and delete once they are uploaded to the secure drive. Written materials such as consent and assent forms will be stored in a locked drawer in a locked office (See confidentiality for more details).

Analysis**.* (Aim 1) Quantitative longitudinal analysis will explore changes in depression and secondary outcomes from baseline to 3-month post-treatment based on intention-to-treat. Descriptive measures will be used to summarize data. Depression changes between baseline and post, 1-month post, and 3-month follow-up will be compared in the LEAD versus WL arms through mixed-effects linear regression models, with fixed effects for study condition, time, and the interaction between condition and time. (Aim 2) Mixed-methods: quantitative and qualitative findings will be equally valued, analyzed separately, then integrated guided by hypothesized change pathways. ***Quantitative: we will explore pathways using structural equation modeling with data from Baseline, During Treatment [proximal mechanisms], Post, 1-month, and 3-month post. We will conduct a path analysis. We will explore 3 pathways: (1) the indirect effect of LEAD on father; (2) the direct and indirect effect of father depression on child MH via parenting, and (3) if we detect relationships in path 1 and 2, we will explore the direct and indirect effects of LEAD on child MH via father's depression. ***Qualitative:*** We will use the Framework Method[29] with inductive and deductive coding[53]. Two RAs will familiarize themselves with interview and session

transcripts. Next, they will open-code transcripts line-by-line, then deductively code transcripts. Once data are

coded, they will be charted and assessed with a framework matrix. Results will clarify and contextualize mechanisms, and potential moderators, to test in a future trial. (Aim 3) Table 3 shows measures for Aim 3.

Interviews and focus groups will be analyzed using the Framework Method. ***Participation*** will be analyzed as % of eligible fathers who enroll in LEAD and % excluded; *retention* will be % of fathers attending ≥ 1 session, attending ≥ 75% of sessions, and dropping out. We will explore retention rates across both arms. To examine ***fidelity*** and ***counselor competency***, all sessions will be audio recorded; 25% will be transcribed, translated to English, and reviewed by the PI and the PC. We will conduct consensus ratings on the first 4 transcripts, then reach 80% agreement on ratings before independently rating the remainder. Fidelity will yield two scores: (1) % of steps completed based on proportion of LEAD components delivered as intended and (2) mean quality scores across steps for each case. ***Counselor competency*** will be calculated as score averages per counselor session.

Table 3. Aim 3 Assessment of Implementation Outcomes

Measure	Reporter	RE-AIM Domain
Acceptability		
Semi-structured interview	F	*Adoption*
Focus Groups	PF, S, CL, H	
Acceptability of Intervention Measure (4-item Survey)	PF, S, CL, H, F	
Intervention Appropriateness Measures (4-item survey)	PF, S, CL, H, F	
Feasibility		
Father participation: % enrolled & % excluded	Tracked	*Reach*
Fidelity Checklist: *Adherence & Delivery Quality*	Coded	*Implementation*
Counselor Competency (ENACT Scale)	Coded	
Retention: Attendance & Attrition	Tracked	
Semi-structured interview	F	
Focus Groups	PF, S, CL, H	
Feasibility of Intervention Measure (4-itme survey)	PF, S, CL, H, F	

PF=Peer-father counselor; S=Supervisor; F=Father; CL=community leaders; H= Hospital Staff; RA's complete assessments; ***Effectiveness Aim 1, 2 focus***.

TIMEFRAME

In Years 1-4, I will conduct the pilot evaluating LEAD compared to a waitlist control (WL; Aims 1-3). Data analysis (Years 3-4) will explore changes in father MH and secondary outcomes (e.g., child MH) between groups (**Aim 1**). I will use mixed methods to explore mechanisms impacting father, family, and child outcomes (**Aim 2**). In Year 4, I will evaluate feasibility and acceptability of implementing LEAD (**Aim 3**) and develop an R01 proposal. In Year 5, I will complete analysis and manuscripts, disseminate findings in the US and Kenya, and submit the R01 proposal.

Timeline	Year 1				Year 2				Year 3				Year 4				Year 5			
**Quarter**	**1**	**2**	**3**	**4**	**1**	**2**	**3**	**4**	**1**	**2**	**3**	**4**	**1**	**2**	**3**	**4**	**1**	**2**	**3**	**4**
**STUDY**: Conduct a pilot randomized trial of Learn, Engage, Act, Dedicate (LEAD), a behavioral activation treatment, compared to a waitlist control to: ***Aim 1*** . Explore the preliminary effctiveness of LEAD; ***Aim 2*** . Use mixed methods to explore mechanisms driving change/no change in father MH, father parenting, and child MH; ***Aim 3*** . Explore feasibility and acceptability of implementing task-shifted MH treatment for fathers in a low-resource setting.																				
Initiate Community Meetings	■																			
IRB Approvals	■																			
Hire, Train RAs (ethics; project goals, methods)	■																			
Train Ennumerators (Assessments) & CHWs (Recruitmen t)	■	■																		
Supervisor Recruitment/Hiring		■																		
Supervisor Training		■																		
Counselor Rectuitment		■	■																	
Counselor Training			■																	
Particpant Recruitment			■	■	■	■	■	■	■	■										
Counselor Refersher Training (2 days)						■		■		■										
Conduct Pilot (Assessment, Treatment/TAU)				■	■	■	■	■	■	■	■	■	■	■						
Aim 1 Data Analysis (Explore Clinical Change)														■	■					
Aim 2 Data Analysis (Mixed-Methods)															■	■				
Aim 3 Data Analysis (Implementation Exploration)																■	■			
Community Dissemination																	■	■		
R01 Development; Publish															■	■	■	■	■	■

Ethics Consideration

Risks

There are no direct physical risks associated with participation in the study. However, there may be psychological and privacy risks. For men, screening and discussions of mental illness in treatment may elicit uncomfortable emotions for patients and counselors. While the aim of LEAD is depression symptom reduction, it is possible participants may experience a worsening of symptoms. All counselors, supervisors, and RAs will be trained to provide counseling in the management of such feelings and may refer participants to specialized care with a psychologist, psychiatrist, or psychologist/psychiatrist in training at MTRH. The on-site PI is also well trained in risk assessment and can provide in the moment care and triage as need. Further, for men who are excluded from the study due to severe problems, referrals and appointments to care at MTRH will be made by the consenters. With permission, referrals will also be given to men who are eligible but do not consent to the study. For co-caregivers and children, mental health screening may elicit uncomfortable emotions as well. It is also possible that participants and reporters (co-caregivers, youth) may report abuse or suicidal ideation during screening or, for men, during treatment. It is also possible co-caregiver or youth participating in assessments may

place them at risk of violence in the home, though a host of precautions will be taken to minimize this risk. A careful protocol has been developed outlining the responsibilities of the research staff to ensure coordination of management strategies that best meet participants' psychiatric/medical needs and well-being; this is based on practices we have used previously in the context of family therapy and substance use and depression treatments in Eldoret. Additionally, for lay counselors, it is possible that thinking about the issues related to the implementation process may be mildly upsetting during post-treatment interviews, but this is expected to be transient if it occurs. Individual identifiers of participants will not be collected. However, to limit threats to breach of confidentiality, several steps will be taken to secure digital and paper-based information (See above).

Benefits

Fathers in the treatment arm will have the opportunity to receive a free, evidence-based intervention in which they will learn skills to manage their symptoms and strategies to address potential sources of distress. All screened will be referred to care. There is also the potential that men's family interactions may improve, and in turn family and child outcomes may improve. As such there may be some potential benefit for children and co-caregivers. Through assessment, participants from both study arms will have the opportunity to reflect on issues and treatment impact, which may be empowering and increase self-awareness. Assessments will be used to inform future services that they may benefit directly from (participants) or that may facilitate their treatment delivery (lay counselors, delivery stakeholders). Findings from this study may be applied to other low-resource settings to improve services for the management of men's depression with potential positive impact on family and child outcomes. Lay counselors and supervisors will receive free training on treatment, including in behavioral activation, motivational interviewing, and dealing with clinical emergencies. We hope this can help improve the treatment gap by training new providers to provide mental health care.

Informed Consent

Participants: All pilot RCT participants and co-caregiver reporters will provide written informed consent separately. For all participants, before any data is collected, the CHW, PC, and/or RAs will review the following through the consenting process: 1) purpose, 2) locations where research will be conducted, 3) points of assessment and the kinds of questions that will be asked, 4) description of the study, 5) how privacy will be protected, 6) potential risks, 7) potential benefits, and 8) the participant's rights. For men and co-caregivers, privacy of answers between family members will be emphasized; and men's privacy in treatment will be emphasized. Men's treatment will be described to co-caregivers as a leadership program that helps men reach goals and, sometimes, feel better. For co-caregiver's, their and their

child's safety and comfort with participating in assessments will be emphasized, noting how procedures can be discontinued at any point and the reasons for discontinuing will remain confidential. Extra time will be taken to discuss how answers will be kept confidential and how dummy questions will be used if privacy is ever compromised. The interviewer will read the consent forms and take the extra step of assessing whether a participant's factual understanding of information is sufficient to provide ethically valid consent to participate in the research study. For youth reporters, caregiver (men or co-caregiver) consent will be obtained for child participation and permission to speak with the target child in assessment. Child assent will then be obtained with the study purpose and procedures explained at developmentally appropriate levels. These are procedures that have been previously piloted and were developed with the guidance of mentors and advisors conducting work with vulnerable families in Eldoret and globally.

Mental Health Counselors and Delivery Staff: An information sheet will be read and explained, and if the peer-father counselor or key delivery stakeholder (supervisor, community leader, staff) agrees to participate with a written signature, focus groups will be conducted 1-month after all treatment activities have ceased conducted by an enumerator, RA, or PC. Consent and assent forms will be read aloud to each participant and a written copy will be provided before beginning study participation. They will be offered in both English and Swahili depending on the participants preferred language. The local/onsite PI, Dr. Florence Jaguga, and I will oversee and supervise the research ethics training alongside training in WHO guidelines for ethical research in cases of dealing with violence of research staff who will be obtaining consent and assent.

Confidentiality

We will ensure the following steps to protect confidentiality: 1) all data will be labeled only with study identification numbers, and no participant names will be attached to study data; 2) electronic data files will be password protected and stored on a secure study server, which requires authentication to access; 3) the participant "key" linking study identification numbers with participant names will stored on a secure server, separate from all other study data, to which only I and the Project Coordinator have access; 4) all paper research data (which are only the informed consent and assent forms) will be kept in locked file cabinets in Dr. Jaguga's office in MTRH and will be available only to research staff directly involved in this project; 5) all research staff will receive training on procedures to protect participant confidentiality and will take all required courses and certification tests; and 6) dummy assessment questions will be created in case interviews are interrupted in real time. Participants will be told that all data are confidential within the limits of the law. To address confidentiality of interview audio recordings, I, the PC, and a hired professional transcriber will be the only people permitted to listen to the digital recordings. In addition to those described, session recordings can be accessed once by treatment supervisors following a treatment session; after use for supervision, the recordings will be uploaded and deleted from recorders. All digital recordings will be stored on a secure server at all times

in password protected files. Interviews will be transcribed, without recording names, by a professional transcriber and linked to a numerical identifier. Participants will be informed that audio recording is a requirement of study participation for interviews only. However, participants will be free to withdraw consent/assent at any time. Neither the participants' names nor any personal information identifying the participants will be used in connection with the audio recordings in any way when findings are presented in professional forums or publications.

Ethical approval:

Ethical approval will be sought from the MTRH/ Moi University Institutional Research Ethics committee (IREC) as well as Florida International University (FIU) Institutional review board.

Study Implications:

This study could estimate the effect of a brief, task-shifted intervention to improve father depression and explore potential for in turn improving child mental health. Tested in a future R01 effectiveness-implementation Hybrid trial, this intervention has the potential to engage a hard to engage population (men) and has potential for reach given it is brief and uses task-shifting. It will also produce data on the challenges and facilitators to implementation of depression treatment for men in low-resource settings abroad and in the US. Further this work, in its exploration of mechanisms in analysis, can inform if and by what pathways men's treatment engagement may or may not impact family and child outcomes; this has implications for understanding and refining treatment to better target potential mechanisms. Lastly, this knowledge will be applied to scale-up of services in Kenya and may be used to inform scale-up of interventions for fathers globally, helping to reduce the global mental health treatment gap.

APPENDIX I: CONSENT/ ASSENT FORMS

Father Consent
Informed Consent for Participation in Research
Title of Study: Father 'Learn, Engage, Act, Dedicate' (LEAD) Program RCT

Key Information
1. We are doing a voluntary research project.
2. The goal is to learn about the feasibility and acceptable of a program called LEAD, for fathers are feeling down and who may want to change their drinking. We also want to see if this program helps with these problems and issues in the family. We hope to learn this through your input and the input of others in the community.
3. The risks are minimal, but it is possible some questions might make you uncomfortable.
4. This will help how and if LEAD [Learn, Engage, Act, Dedicate] works for fathers in your community.

Purpose and Overview
The research is being done by researchers from Florida International University (FIU) and Referral Hospital who are working with AMPATH. The purpose is to learn how a program that helps father's feeling low or having trouble reaching their goals find ways to feel better and reach their goals. I would like to tell you about the research and (1) ask if you would like to participate and (2) see if you are eligible to participate. We are including fathers who have children ages 8-17 and are reporting some of these problems like feeling very down and also drinking.

Voluntary
Participation in this research study is voluntary. You do not have to participate if you do not want to. Declining will not affect you in any way. You can also stop at any time and it will not affect any other services or programs provided by AMPATH or any other organization that you participate in or that you may want to participate in in the future.

Procedures (What you will be asked to do)
First, we would start by asking you a few questions to determine if you are eligible to participate in this study. If you are *not* eligible but are reporting mental health issues you will still be referred to MTRH care as usual. If you are eligible and interested in participating, then we will conduct a longer 30 to 45 minute interview with you to ask more questions about your psychological wellbeing, drinking, family relationships, and sense of safety. Depending on your preference, and if it is safe to do so, we will conduct these interviews in person at your home, offices affiliated with MTRH or another private location that is convenient for you. If non-essential in-person activities are not safe due to COVID-19, we will conduct these interviews over the phone or internet.

After the interview, you will be randomized to either receive the LEAD program as soon as possible or wait about 4 months to receive LEAD. Regardless of what group you are selected to, to wait or to LEAD, you will receive referrals to care at MTRH if indicated. If you are randomly selected the LEAD group, you will meet 5 times for around 40-90 minutes with a counselor trained to deliver this intervention at a safe

and private space. Examples of topics that may be covered in the program are identifying values (i.e., what is important to you), tracking your mood, scheduling activities to reach your goals, and how to say no to situations that make you feel down or low. At the end of LEAD, we will repeat the 30-45 minute interview 3 times: once right after finishing, once 1 month after, and once 3 months after. One or three months after LEAD, we may also ask you if you are interested in participating in a longer interview lasting around 1 hour about LEAD, your counselor, and changes you did or did not see.

If you are in the other group, you will complete these same interviews minus the longer 1 hour interview. The first interview will be about 5 or 6 weeks from now. Then after your last interview, you will be offered the LEAD program.

Audio Recording: If you are in the LEAD group, some sessions and the 1 hour interviews will be audio recorded. Your name will not be included on anything, so your answers will stay private. Only people on the research team will hear the tapes, once they are transcribed, they are deleted (within a year). You can withdraw your consent at any time and the tape can be destroyed. Recording is *not* required for participation.

Risks and Inconveniences

During the interview we will ask you personal questions about your wellbeing, including some questions about difficulties such as psychological distress. You can stop answering these questions at any time. If you become distressed or uncomfortable during the interview, we will connect you with individuals who are trained to handle these types of issues. If you take part in the intervention, it is possible that during the sessions you may talk about things that make you uncomfortable. The intervention counselors are here to help you if you start to feel this way. Also, counselors and supervisors who are working on this study have received training on how to keep what is said safe and to promote confidentiality among the group members.

Anticipated Benefits
You will receive a one-on-one intervention for free.

Confidentiality
Every effort will be made to keep your personal information confidential. However, we cannot guarantee total privacy or confidentiality. All information that you give us will be kept in a very safe place, locked in a cabinet at our offices and/or on a password-protected computer. We will collect personally identifiable information from you including your name, age, and telephone number in order to schedule interviews and intervention sessions. This information will be kept private and separate from all other information you provide during the interviews and intervention sessions. Only a few people will have access to your personally identifiable information and no one else will be able to see this information. All interview and intervention materials will only include a code and will not include your personally identifiable information. All of your identifying information (name, phone number) will be destroyed when the study is complete. After removal of all personally identifiable information, the data could be used for future research studies or distributed to another investigator for future research without additional informed consent. If information from this study is published or presented at meetings, your name and other personal information about you will not be used. De-identified records will be available to research staff, and to Federal, State and Institutional regulatory personnel (who may review records as part of routine audits).

A description of this clinical trial will be available on ClinicalTrials.gov, as required by US Law. This web site will not include information that can identify you. At most, the web site will include a summary of the results. You can search this website at anytime

Study Compensation

You will be provided compensation to complete interviews. Travel will be reimbursed. If you complete the interviews remotely we will reimburse you for your airtime or data. We will not pay you for your participation.

In Case of Injury

If you believe that you have sustained an injury as a result of participating in a research study, you may contact the Principal Investigator at +254 726 626391 or +019546145393 so that you can review the matter and identify the medical resources that may be available to you.

Questions

If you have any questions or worries about the study, you can contact Florence Jaguga (+254 726 626391) or Ali Giusto, the lead researcher. If you have questions about the rights of people who participate in research studies, Dr. Jaguga or Dr. Giusto will contact the board at AMPATH that is in charge of the ethics of conducting research. If you have any questions about your rights as a research participant, want to provide feedback, or have a complaint, you may you may contact the FIU Office of Research Integrity by phone at 305-348-2494 or by email at ori@fiu.edu. (An IRB is a committee that protects the rights of participants in research studies).

Documentation of Consent & Participant Agreement
Nyaraka ya Ridhaa

PARTICIPANT AGREEMENT

I have read the information in this consent form and agree to participate in this study. I have had a chance to ask any questions I have about this study, and they have been answered for me. I understand that I will be given a copy of this form for my records if I would like.

Do you voluntarily agree to participate in this study?
Je, unakubali kushiriki katika utafiti huu?

Date: ________________________

Print Name: ___________________

Signature of Participant:
Sahihi ya mshiriki:

I have discussed the proposed research with this participant including the risks, benefits, and alternatives to participation (including the alternative of not participating in the research). The participant has had an opportunity to ask questions and in my opinion is capable of freely consenting to participate in this research.

Print name: __________________
Person Designated to Obtain Consent Signed: _____________________
Date: ______________________

Partner Consent: Focus Group Discussion

Informed Consent for Participation in Research

Title of Study: Father 'Learn, Engage, Act, Dedicate' (LEAD) Program RCT

Key Information

1. We are doing a voluntary research project.
2. We are doing a project to learn about mental health and family relationships like how families get along in your community. This will help us learn more about programs for individuals in the family that may help not only the person but the whole family. We hope to learn this through your input and the input of others in the community.
3. The risks are minimal, but it is possible some questions might make you uncomfortable.
4. This will help us understand how programs family members do or do not influence families.

Purpose and Overview

The research is being done by researchers from Florida International University (FIU) and Moi Teaching and Referral Hospital who are working with AMPATH. The purpose of this is to understand more about female caregiver and youth psychological wellbeing, relationships in the family, and perspectives on male caregiver family involvement. I would like to tell you about the research and (1) ask if you would like to participate and (2) see if you are eligible to participate. We are including fathers who have children ages 8-17 and are reporting some of these problems like feeling very down and also drinking.

Voluntary

Participation in this research study is voluntary. You do not have to participate if you do not want to. Declining will not affect you in any way. You can also stop at any time and it will not affect any other services or programs provided by AMPATH or any other organization that you participate in or that you may want to participate in in the future.

Procedures

First, we would start by asking you a few questions to determine if you are eligible to participate in this study: this means you feel comfortable participating without any fear and you have a child between the ages of 8-17 who may be experiencing some psychological distress. If you are eligible and interested in participating, we will conduct a 30 minute interview with you to ask more questions about your psychological wellbeing, family relationships, and male caregiver family interactions. Depending on your preference, and if it is safe to do so, we will conduct these interviews in person at your home, offices affiliated with MTRH or another private location that is convenient for you. If non-essential in-person activities are not safe due to COVID-19, we will conduct these interviews over the phone or internet. *We want to emphasize that these activities are voluntary, and every effort will be made to keep your answers private and that interviews will be conducted where others cannot hear. If you feel any discomfort answer questions specifically about male caregiver behavior or you fear that doing so would put you at risk, then the interview can be discontinued, and your participation withdrawn. No information will be shared with your partner.* After the first interview, three more 30 minute interviews will be conducted: one around 5-6 weeks after the initial interview, then one a month after that, and one three months after that. We may also ask you if you are interested in participating in a longer interview lasting around 1 hour your well being and family relationships.

Audio Recording: If you complete a longer 1-hour interview this will be audio recorded; your name will not be included on anything. Your name will not be included on anything, so your answers will stay private. Only people on the research team will hear the tapes, once they are transcribed, they are deleted (within a year). You can withdraw your consent at any time and the tape can be destroyed. Recording is *not* required for participation.

Risks and Inconveniences

During the interview we will ask you personal questions about your wellbeing, including some questions about difficulties such as psychological distress. You can stop answering these questions at any time. If you become distressed or uncomfortable during the interview, we will connect you with individuals who are trained to handle these types of issues. There is a slight possibility someone may walk in to an ongoing interview, if this happens, we have prepared "dummy" questions about innocuous topics like food that the interview can ask in the off chance an interview is interrupted by another individual.

Anticipated Benefits

You will not benefit directly from participating. You will not receive any services. However, you will be helping us learn information that will help us understand programs for people in your community in the future and about families.

Confidentiality:

Every effort will be made to keep your personal information confidential. However, we cannot guarantee total privacy or confidentiality. All information that you give us will be kept in a very safe place, locked in a cabinet at our offices and/or on a password-protected computer. We will collect personally identifiable information from you including your name, age, and telephone number in order to schedule interviews and intervention sessions. This information will be kept private and separate from all other information you provide during the interviews and intervention sessions. Only a few people will have access to your personally identifiable information and no one else will be able to see this information. All interview and intervention materials will only include a code and will not include your personally identifiable information. All of your identifying information (name, phone number) will be destroyed when the study is complete. After removal of all personally identifiable information, the data could be used for future research studies or distributed to another investigator for future research without additional informed consent. If information from this study is published or presented at meetings, your name and other personal information about you will not be used. De-identified records will be available to research staff, and to Federal, State and Institutional regulatory personnel (who may review records as part of routine audits). These is also a chance someone's responses may indicate they are in danger. We do not expect this to happen, but if it does, then the interviewer would let a trained supervisor know in order to keep you safe. A description of this clinical trial will be available on ClinicalTrials.gov, as required by US Law. This web site will not include information that can identify you. At most, the web site will include a summary of the results. You can search this website at anytime

Study Compensation

You will be provided compensation to complete interviews. Travel will be reimbursed. If you complete the interviews remotely we will reimburse you for your airtime or data. We will not pay you for your participation.

In Case of Injury

If you believe that you have sustained an injury as a result of participating in a research study, you may contact the Principal Investigator at +254 726 626391 or +019546145393 so that you can review the matter and identify the medical resources that may be available to you.

Questions
If you have any questions or worries about the study, you can contact Florence Jaguga (+254 726 626391) or Ali Giusto, the lead researcher. If you have questions about the rights of people who participate in research studies, Dr. Jaguga or Dr. Giusto will contact the board at AMPATH that is in charge of the ethics of conducting research. If you have any questions about your rights as a research participant, want to provide feedback, or have a complaint, you may you may contact the FIU Office of Research Integrity by phone at 305-348-2494 or by email at ori@fiu.edu. (An IRB is a committee that protects the rights of participants in research studies).

Documentation of Consent & Participant Agreement
Nyaraka ya Ridhaa

PARTICIPANT AGREEMENT

I have read the information in this consent form and agree to participate in this study. I have had a chance to ask any questions I have about this study, and they have been answered for me. I understand that I will be given a copy of this form for my records if I would like.

Do you voluntarily agree to participate in this study?
Je, unakubali kushiriki katika utafiti huu?

Date: ________________________

Print Name: ___________________

Signature of Participant:
Sahihi ya mshiriki:
____

I have discussed the proposed research with this participant including the risks, benefits, and alternatives to participation (including the alternative of not participating in the research). The participant has had an opportunity to ask questions and in my opinion is capable of freely consenting to participate in this research.
Print name: __________________
Person Designated to Obtain Consent Signed: _____________________
Date: _______________________

Caregiver Permission for Youth Participation
Title of Study: Father 'Learn, Engage, Act, Dedicate' (LEAD) Program RCT

Key Information
1. We are doing a voluntary research project.
2. We are doing a project to learn about mental health and family relationships like how families get along. This will help us learn more about programs for individuals in the family that may help the whole family. We hope to learn this through your input and the input of others in the community.
3. The risks are minimal, but it is possible some questions might make you uncomfortable.
4. This will help us understand youth and family well-being in your community.

Purpose and Overview
The research is being done by researchers from Florida International University (FIU) and Moi Teaching and Referral Hospital who are working with AMPATH. The purpose is to learn how a program that helps father's feeling low or having trouble reaching their goals find ways to feel better and reach their goals. I would like to tell you about the research and (1) invite one of your children to participate if you have decided to participate. Youth who are eligible are those who have a male caregiver participating in the research, is between the ages of 8-17, and is experiencing some psychological distress as reported by the youth or the female or male caregiver.

Voluntary
Participation in this research study is voluntary. You do not have to participate if you do not want to. Saying no will not affect careers or patient care in any way. You can also stop at any time and it will not affect any other services or programs provided by AMPATH or any other organization that you might participate in or that you may want to participate in in the future.

Procedures
We would like to invite your child to participate in survey sessions to help with the research. First, if you decide to participate, one child will be asked to meet with one or two researchers to take a 30 minute interview survey. They will be asked questions about their psychological well-being and family relationships. They would complete these interviews four times: once in the next week or so, then in about 5 weeks from that time, one month after that, and three months after that. Interviews will be done in a private space at home, at MTRH offices (accompanied by a caregiver), or another private location. The interviews are done to understand how to help families.

Audio Recording: The interviews may be audio recorded so that the researchers can learn more about how to help engage men in the program, but your name will not be included on anything, so your answers will stay private. Only people on the research team will hear the tapes, once they are transcribed, they are deleted (within a year). You can withdraw your consent at any time and the tape can be destroyed. Recording is required for participation.

Risks and Inconveniences
During the interview we will ask your child some personal questions about their wellbeing, including some questions about difficulties such as psychological distress. They can stop answering these questions at any time, and the interviewers are trained to work with youth and detect any signals of distress. If they become distressed or uncomfortable during the interview, we will connect them with individuals who are trained to handle these types of issues.

Anticipated Benefits

They will not benefit directly from participating. They will not receive any services. However, they will be helping us learn information to help families in the future.

Confidentiality

Every effort will be made to keep their personal information confidential. However, we cannot guarantee total privacy or confidentiality. All information that your child gives us will be kept in a very safe place, locked in a cabinet at our offices and/or on a password-protected computer. We will collect personally identifiable information (name, age) to help schedule interviews. This information will be kept private and separate from all other information they provide during the interviews. Only a few people will have access to personally identifiable information and no one else will be able to see this information. All interview materials will only include a code and will not include your personally identifiable information. All identifying information (name, phone number) will be destroyed when the study is complete. After removal of all personally identifiable information, the data could be used for future research studies or distributed to another investigator for future research without additional informed consent. If information from this study is published or presented at meetings, their name and other personal information will not be used. De-identified records will be available to research staff, and to Federal, State and Institutional regulatory personnel (who may review records as part of routine audits). These is also a chance someone's responses may indicate they are in danger. We do not expect this to happen, but if it does, then the interviewer would let a trained supervisor know in order to keep the youth safe and you or another caregiver would be informed. A description of this clinical trial will be available on ClinicalTrials.gov, as required by US Law. This web site will not include information that can identify you. At most, the web site will include a summary of the results. You can search this website at anytime

Study Compensation

Youth will receive a small token worth around ~100 KSH such as a notebook or pens for participating. Travel will be reimbursed for caregivers who might accompany youth to an interview.

In Case of Injury

If you believe that you have sustained an injury as a result of participating in a research study, you may contact the Principal Investigator at +254 726 626391 or +019546145393 so that you can review the matter and identify the medical resources that may be available to you.

Questions

If you have any questions or worries about the study, you can contact Florence Jaguga (+254 726 626391) or Ali Giusto, the lead researcher. If you have questions about the rights of people who participate in research studies, Dr. Jaguga or Dr. Giusto will contact the board at AMPATH that is in charge of the ethics of conducting research. If you have any questions about your rights as a research participant, want to provide feedback, or have a complaint, you may you may contact the FIU Office of Research Integrity by phone at 305-348-2494 or by email at ori@fiu.edu. (An IRB is a committee that protects the rights of participants in research studies).

Documentation of Consent & Participant Agreement
Nyaraka ya Ridhaa

PARTICIPANT AGREEMENT

I have read the information in this consent form and agree to participate in this study. I have had a chance to ask any questions I have about this study, and they have been answered for me. I understand that I will be given a copy of this form for my records if I would like.

Do you voluntarily agree to allow your child to participate in this study?
Je, unakubali kushiriki katika utafiti huu?

Date: ________________________

Print Name: ___________________

Signature of Caregiver:
Sahihi ya mshiriki:
___

I have discussed the proposed research with this participant including the risks, benefits, and alternatives to participation (including the alternative of not participating in the research). The participant has had an opportunity to ask questions and in my opinion is capable of freely consenting to participate in this research.
Print name: __________________
Person Designated to Obtain Permission Signed: _____________________
Date: _______________________

Implementation Stakeholder Consent (Providers/Supervisors/Staff)
Informed Consent for Participation in Research
Title of Study: Father 'Learn, Engage, Act, Dedicate' (LEAD) Program RCT

Key Information
1. This is a voluntary research project.
2. The goal is to learn about how feasible and acceptable LEAD is for fathers who want to change their drinking and those delivering the program. We hope to learn this through an interview and some survey questions with you that will last around one hour.
3. The risks are minimal.
4. This will help us understand if the program is acceptable and feasible.

Purpose and Overview
The research is being done by researchers from Florida International University (FIU) and Moi Teaching and Referral Hospital who are working with AMPATH. The purpose of the overall study is to learn if LEAD helps father's feeling low or having trouble reaching their goals find ways to feel better and reach their goals. I would like to tell you about the research and ask if you would like to participate in an interview.

Voluntary
Participation in this research study is voluntary. You do not have to participate if you do not want to. Declining will not affect you in any way.

Procedures
We would like you to invite you to participate in an interview with a member of our research staff. If you decide to participate, you will be asked to discuss things like how satisfied you were with program delivery, how acceptable the program and its delivery was, and fill out survey questions on the same topics.

Audio Recording: The interviews will be audio recorded so that we can learn more about the program's acceptability and feasibility. Your name will not be included on anything, so your answers will stay private. Only people on the research team will hear the tapes, once they are transcribed, they are deleted (within a year). You can withdraw your consent at any time and the tape can be destroyed. Recording is *not* required for participation.

Risks and Inconveniences
The risks are minimal. If we ask you a question that you do not want to answer, then you do not have to answer. We will keep the recordings locked on our computers. However, there is always the small possibility that someone else might hear the discussion.

Anticipated Benefits
You will not benefit directly from participating. However, you will be helping us learn information that will help us develop ways to deliver programs for people in your community in the future.

Confidentiality

Every effort will be made to keep your personal information confidential. However, we cannot guarantee total privacy or confidentiality. All information that you give us will be kept in a very safe place, locked in a cabinet at our offices and/or on a password-protected computer. We will collect personally identifiable information from you including your name, age, and telephone number in order to schedule interviews and intervention sessions. This information will be kept private and separate from all other information you provide during the interviews and intervention sessions. Only a few people will have access to your personally identifiable information and no one else will be able to see this information. All interview will only include a code and will not include your personally identifiable information. All of your identifying information (name, phone number) will be destroyed when the study is complete. After removal of all personally identifiable information, the data could be used for future research studies or distributed to another investigator for future research without additional informed consent. If information from this study is published or presented at meetings, your name and other personal information about you will not be used. De-identified records will be available to research staff, and to Federal, State and Institutional regulatory personnel (who may review records as part of routine audits). A description of this clinical trial will be available on ClinicalTrials.gov, as required by US Law. This web site will not include information that can identify you. At most, the web site will include a summary of the results. You can search this website at anytime

Study Compensation

Any travel to the interview will be reimbursed/or airtime will be reimbursed if the interview is conducted over the phone. You will be compensated for completing the interview.

In Case of Injury

If you believe that you have sustained an injury as a result of participating in a research study, you may contact the Principal Investigator at +254 726 626391 or +019546145393 so that you can review the matter and identify the medical resources that may be available to you.

Questions

If you have any questions or worries about the study, you can contact Florence Jaguga (+254 726 626391) or Ali Giusto, the lead researcher. If you have questions about the rights of people who participate in research studies, Dr. Jaguga or Dr. Giusto will contact the board at AMPATH that is in charge of the ethics of conducting research. If you have any questions about your rights as a research participant, want to provide feedback, or have a complaint, you may you may contact the FIU Office of Research Integrity by phone at 305-348-2494 or by email at ori@fiu.edu. (An IRB is a committee that protects the rights of participants in research studies).

Documentation of Consent & Participant Agreement
Nyaraka ya Ridhaa

PARTICIPANT AGREEMENT

I have read the information in this consent form and agree to participate in this study. I have had a chance to ask any questions I have about this study, and they have been answered for me. I understand that I will be given a copy of this form for my records if I would like.

Do you voluntarily agree to participate in this study?
Je, unakubali kushiriki katika utafiti huu?

Date: ________________________

Signature of Participant:
Sahihi ya mshiriki:

I have discussed the proposed research with this participant including the risks, benefits, and alternatives to participation (including the alternative of not participating in the research). The participant has had an opportunity to ask questions and in my opinion is capable of freely consenting to participate in this research.

Print name: ________________

Person Designated to Obtain Consent Signed: ____________________

Date: _____________________

APPENDIX II: Examples of Questionnaires/Surveys

<u>Sociodemographic Question Examples</u>:

Patient Study Number:………………….. Date of recruitment:…………………………….

Date of birth……………. Age………………………..Mobile no……………………………….

Gender

Male ☐ Female ☐ Other ☐

Level of education

No Primary ☐

Incomplete primary ☐

Complete primary ☐

Incomplete secondary ☐

Complete secondary ☐

Tertiary + ☐

Marital status

Never married ☐ Separated/divorced/widowed ☐ Married/cohabiting ☐

Living arrangement; Lives with

V

Family/relative ☐ friend/non-relative ☐ Alone ☐

What is your occupation or what you do to make money? _________________________

How many children do you have? ________________

What is the age of the oldest and youngest?_____________

Example PHQ-9 Questionnaire:

**Over the last 2 weeks, how often have you been bothered by any of the following problems?** *(Use "✔" to indicate your answer")*	**Never**	**1-3 days in a week**	**4-5 days in a week**	**6-7 days in a week**
**1. Little interest or pleasure in doing things...............**	**0**	**1**	**2**	**3**
**2. Feeling down, depressed, or hopeless.................**	**0**	**1**	**2**	**3**
3. Trouble falling or staying asleep, or sleeping too much..	0	1	2	3
4. Feeling tired or having little energy......................	0	1	2	3
5. Poor appetite or overeating..................................	0	1	2	3
6. Feeling bad about yourself — or that you are a failure or have let yourself or your family down.....................	0	1	2	3
7. Trouble concentrating on thing s, such as reading the newspaper or watching television...........................	0	1	2	3
8. Moving or speaking so slowly that other people could have noticed? Or the opposite — being so fidgety or restless that you have been moving .around a lot more than usual..	0	1	2	3
9. Thoughts that you would be better off dead or of hurting yourself in some way..	0	1	2	3

Drinking Question Examples (Alcohol Use Disorder Identification Test; AUDIT)

Please circle the answer that is correct for you

1. How often do you have a drink containing alcohol? · Never · Monthly or less · 2-4 times a month · 2-3 times a week · 4 or more times a week

2. How many standard drinks containing alcohol do you have on a typical day when drinking? · 1 or 2 · 3 or 4 · 5 or 6 · 7 to 9 · 10 or more

V

3. How often do you have six or more drinks on one occasion? · Never · Less than monthly · Monthly · Weekly · Daily or almost daily
4. During the past year, how often have you found that you were not able to stop drinking once you had started? · Never · Less than monthly · Monthly · Weekly · Daily or almost daily

[Pre_CAGE-AID_ Screener] Do you use any substances other than alcohol such as cigarettes, khat, and cannabis? Yes$_0$ No$_1$
IF YES, ask the remaining CAGE-AID questions. IF NO, skip to the next section.
CAGE1. Have you ever felt the need to cut down on your drug use? Yes$_0$ No$_1$
CAGE2. Have people annoyed you by criticizing your drug use? Yes$_0$ No$_1$
CAGE3. Have you ever felt guilty about drug use? Yes$_0$ No$_1$
CAGE4. Have you ever felt you needed to use drugs first thing in the morning to steady your nerves or to get rid of a hangover? Yes$_0$ No$_1$

Example Family Relationship Questions:

Parenting

1. I make it easy for my child to confide in me
2. I am harsh with my child
3. I enjoy having my child around me
4. I make my child feel proud when (s)he does well
5. I praise my child to others
6. I nag or scold my child when (s)he is bad
7. I punish my child when I am angry

Couple Relationship

1. Over the past two months how often have you and your spouse treated one another with kindness?
2. In the past two months, how much have you disagreed or agreed on...One person in the relationship keeping money to himself
3. Over the past two months has there been understanding between you and your spouse?
4. Over the past two months how often have your and your spouse talked openly about money?
5. Over the past two months how often have you and your spouse decided together how to spend your money?

Joint Parenting

1. My child's other parent and I have the same level of responsibility in disciplining our child.
2. My child's other parent is a good role model for our child.
3. My child's other parent spends enough time with our child.
4. My child's other parent is a good advisor to our child.

Child Mental Health Examples:

1. I try to be nice to other people. I care about their feelings
2. I am restless, I cannot stay still for long
3. I get a lot of headaches, stomach-aches or sickness
4. I usually share with others, for example CD's, games, food
5. I get very angry and often lose my temper
6. I would rather be alone than with people of my age

Implementation Acceptability and Feasibility Survey Questions:

Acceptability of Intervention Measure
1) The program meets my approval.
2) The program is appealing to me.
3) I like the program.
4) I welcome the program.

Intervention Appropriateness Measure (IAM)
1) The program seems fitting.
2) The program seems suitable.
3) The program seems applicable.
4) The program seems like a good match.

Feasibility of Intervention Measure (FIM)
1) The program seems implementable.
2) The program seems possible.
3) The program seems doable.
4) The program seems easy to use.

Qualitative Interview Guide Question Examples:

Fathers

1. Think back to before you started the program, tell me about why you agreed to do this program.
    - Probes:
        - What did you think the counseling would be like?
        - How did you think it would help?
        - Were there things you were confused about or worried about related to starting?
2. Please describe for me what you and the counselor did during the meetings.
    - Probes:
        - What did you talk about?
        - What did they ask you to do?
        - Were you surprised about anything that you did in the sessions?
        - What did you think was most helpful?
        - What did you think of the path?
        - What did you think of planning activities?

3. Some people find changes happen others do not, tell me about if things changes for you and why or why not?
    - What were the problems you wanted help with?
    - Did the program help with these problems?
        - What were positive changes? (get specific examples)
            - Tell me about how your well-being changed?
            - Tell me about how relationships at home. Did they change? Explain
            - Other changes in different parts of your life (e.g., economic changes)?

        Were there things that actually made your feel worse because of the program? (ask for specific examples)
        - What were some problems that the counseling did not help with? (ask for specific examples)
        -
4. How did you feel about the sessions, such as length, location?

Implementation Stakeholders

1. Tell me about your experience delivering this program?
- What were barriers?
- What worked well?

2. Tell me about the content of the intervention?
- What was challenging?
- What worked well?

3. Tell me about the challenges and successes with supervision?
- What would you change?
- What would stay the same?

APPENDIX 10: VERBAL RECRUITMENT SCRIPT

My name is [Name of Research Assistant], and I am a Research Assistant (or Community Health Worker) with Moi Teaching & Referral Hospital clinic. I would like to invite you to participate in a research study whose purpose is to help us understand whether a program for father's wellbeing to help them reach their goals and feel better works in this community.

First, we would like your permission to ask you questions. Those questions will tell us if we think this program would be a good fit for you. These questions can help us know whether our program will be helpful for you or if another program would better to help you reach your goals. If you consent to answering question, then I can let you know if we think this program can help or if we think another program or person could better help. If you are eligible, then you will be randomized to either receive a free leadership and counseling program to help men reach their goals such as reducing drinking or feeling better right away or wait a few months to receive the program. Either way we will still refer you to services in the area if needed (without waiting).

If you consent and are invited to the project, then we will ask your partner/spouse and one of your children if they are between ages 8-17 to be part of the research. **They will not be asked to join the program sessions with you** but will be asked general questions to about the family. Just as your answers to questions will be private from them, theirs will be private from you. Also, they will not be told the type of program that is being provided, though you are welcome to tell them.

If you would like to participate in this research study, I will conduct the screening now (OR refer you to an RA or community health worker to conduct the screening). Do you have any questions now? If you have questions later, please contact the onsite PI at 0726626391 or you may contact 0700131666

Reference:

1. James, S. L. *et al.* Global, regional, and national incidence, prevalence, and years lived with disability for 354 diseases and injuries for 195 countries and territories, 1990–2017: a systematic analysis for the Global Burden of Disease Study 2017. *The Lancet* **392**, 1789–1858 (2018).
2. Wang, P. S. *et al.* Use of mental health services for anxiety, mood, and substance disorders in 17 countries in the WHO world mental health surveys. *The Lancet* **370**, 841–850 (2007).
3. Panter-Brick, C. *et al.* Practitioner Review: Engaging fathers - recommendations for a game change in parenting interventions based on a systematic review of the global evidence. *Journal of Child Psychology and Psychiatry* **55**, 1187–1212 (2014).
4. Seidler, Z. E., Rice, S. M., Ogrodniczuk, J. S., Oliffe, J. L. & Dhillon, H. M. Engaging Men in Psychological Treatment: A Scoping Review. *Am J Mens Health* **12**, 1882–1900 (2018).
5. Seidler, Z. E., Rice, S. M., River, J., Oliffe, J. L. & Dhillon, H. M. Men's Mental Health Services: The Case for a Masculinities Model. *The Journal of Men's Studies* **26**, 92–104 (2018).
6. Wilson, S. & Durbin, C. E. Effects of paternal depression on fathers' parenting behaviors: A meta-analytic review. *Clinical Psychology Review* **30**, 167–180 (2010).
7. Ramchandani, P. G. *et al.* Paternal depression: an examination of its links with father, child and family functioning in the postnatal period. *Depression and Anxiety* **28**, 471–477 (2011).
8. Cabrera, N. J., Fagan, J., Wight, V. & Schadler, C. Influence of Mother, Father, and Child Risk on Parenting and Children's Cognitive and Social Behaviors: Influence of Mother, Father, and Child Risk. *Child Development* **82**, 1985–2005 (2011).

9. Solis, J. M., Shadur, J. M., Burns, A. R. & Hussong, A. M. Understanding the diverse needs of children whose parents abuse substances. *Current drug abuse reviews* **5**, 135 (2012).

10. Leonard, K. E. & Eiden, R. D. Marital and Family Processes in the Context of Alcohol Use and Alcohol Disorders. *Annual Review of Clinical Psychology* **3**, 285–310 (2007).

11. Cuijpers, P., Weitz, E., Karyotaki, E., Garber, J. & Andersson, G. The effects of psychological treatment of maternal depression on children and parental functioning: a meta-analysis. *Eur Child Adolesc Psychiatry* **24**, 237–245 (2015).

12. Erskine, H. E. *et al.* A heavy burden on young minds: the global burden of mental and substance use disorders in children and youth. *Psychol Med* **45**, 1551–1563 (2015).

13. Vogel, D. L., Heimerdinger-Edwards, S. R., Hammer, J. H. & Hubbard, A. “Boys don’t cry”: Examination of the links between endorsement of masculine norms, self-stigma, and help-seeking attitudes for men from diverse backgrounds. *Journal of Counseling Psychology* **58**, 368 (20110530).

14. Bruwer, B. *et al.* Barriers to Mental Health Care and Predictors of Treatment Dropout in the South African Stress and Health Study. **62**, 8 (2011).

15. Wong, J., Ho, M.-H. R., Wang, S.-Y. & Miller, K. Meta-Analyses of the Relationship Between Conformity to Masculine Norms and Mental Health-Related Outcomes. *Journal of Counseling Psychology* (2016).

16. Jewkes, R. K., Dunkle, K., Nduna, M. & Shai, N. Intimate partner violence, relationship power inequity, and incidence of HIV infection in young women in South Africa: a cohort study. *The Lancet* **376**, 41–48 (2010).

17. Masarik, A. S. & Conger, R. D. Stress and child development: a review of the Family Stress Model. *Current Opinion in Psychology* **13**, 85–90 (2017).
18. Giusto, A., Ayuku, D. & Puffer, E. *An intervention to reduce alcohol use and improve family engagement for fathers in low-resource settings: Development and feasibility testing in Kenya*. https://osf.io/wbyct (2020) doi:10.31219/osf.io/wbyct.
19. Kuehn, B. M. Men Face Barriers to Mental Health Care. *JAMA* **296**, 2303 (2006).
20. Kanter, J. W. *et al.* What is behavioral activation? A review of the empirical literature. *Clinical psychology review* (2010) doi:10.1016/j.cpr.2010.04.001.
21. Daughters *et al.* Effectiveness of a brief behavioral treatment for inner-city illicit drug users with elevated depressive symptoms: the life enhancement treatment for substance use (LETS Act!). *Journal of clinical psychiatry* **69**, 122 (2008).
22. Lundahl, B. *et al.* Motivational interviewing in medical care settings: A systematic review and meta-analysis of randomized controlled trials. *Patient Education and Counseling* **93**, 157–168 (2013).
23. Romano, M. & Peters, L. Evaluating the mechanisms of change in motivational interviewing in the treatment of mental health problems: A review and meta-analysis. *Clinical Psychology Review* **38**, 1–12 (2015).
24. Czajkowski, S. M. *et al.* From Ideas to Efficacy: The ORBIT Model for Developing Behavioral Treatments for Chronic Diseases. *Health Psychol* **34**, 971–982 (2015).
25. Giusto, A. *et al.* A multiple baseline study of a brief alcohol reduction and family engagement intervention for fathers in Kenya. *J Consult Clin Psychol* **88**, 708–725 (2020).

26. Leon, A. C., Davis, L. L. & Kraemer, H. C. The role and interpretation of pilot studies in clinical research. *Journal of psychiatric research* **45**, 626–629 (2011).
27. Glasgow, R. E., Battaglia, C., McCreight, M., Ayele, R. A. & Rabin, B. A. Making implementation science more rapid: Use of the RE-AIM framework for mid-course adaptations across five health services research projects in the Veterans Health Administration. *Frontiers in Public Health* **8**, 194 (2020).
28. Billingham, S. A., Whitehead, A. L. & Julious, S. A. An audit of sample sizes for pilot and feasibility trials being undertaken in the United Kingdom registered in the United Kingdom Clinical Research Network database. *BMC Med Res Methodol* **13**, 104 (2013).
29. Gale, N. K., Heath, G., Cameron, E., Rashid, S. & Redwood, S. Using the framework method for the analysis of qualitative data in multi-disciplinary health research. *BMC Medical Research Methodology* **13**, 117 (2013).
30. Hunt, M., Auriemma, J. & Cashaw, A. C. Self-report bias and underreporting of depression on the BDI-II. *Journal of personality assessment* **80**, 26–30 (2003).
31. Vreeman, R. C. *et al.* Characteristics of HIV-infected adolescents enrolled in a disclosure intervention trial in western Kenya. *AIDS Care* **27**, 6–17 (2015).
32. Puffer, E. S. *et al.* A church-based intervention for families to promote mental health and prevent HIV among adolescents in rural Kenya: Results of a randomized trial. *Journal of Consulting and Clinical Psychology* **84**, 511–525 (2016).
33. Monahan, P. O. *et al.* Validity/Reliability of PHQ-9 and PHQ-2 Depression Scales Among Adults Living with HIV/AIDS in Western Kenya. *Journal of General Internal Medicine* **24**, 189–197 (2009).

34. Goodman, R. Psychometric properties of the strengths and difficulties questionnaire. *Journal of the American Academy of Child & Adolescent Psychiatry* **40**, 1337–1345 (2001).

35. Babor, T. F., Higgins-Biddle, J. C., Saunders, J. B. & Monteiro, M. G. Audit. *The Alcohol Use Disorders Identification Test (AUDIT): guidelines for use in primary care* (2001).

36. *Ethical and safety recommendations for intervention research on violence against women. Building on lessons from the WHO publication .Putting women first: ethical and safety recommendations for research on domestic violence against women*. (2016).

37. *Responding to intimate partner violence and sexual violence against women: WHO clinical and policy guidelines.* (2013).

38. Barnett, M. L., Gonzalez, A., Miranda, J., Chavira, D. A. & Lau, A. S. Mobilizing community health workers to address mental health disparities for underserved populations: A systematic review. *Adm Policy Ment Health* **45**, 195–211 (2018).

39. Nadkarni, A. *et al.* Counselling for Alcohol Problems (CAP), a lay counsellor-delivered brief psychological treatment for harmful drinking in men, in primary care in India: a randomised controlled trial. *The Lancet* **389**, 186–195 (2017).

40. Murray, L. K. *et al.* Building capacity in mental health interventions in low resource countries: an apprenticeship model for training local providers. *International journal of mental health systems* **5**, 1–12 (2011).

41. Cuijpers, P., van Straten, A. & Warmerdam, L. Behavioral activation treatments of depression: A meta-analysis. *Clinical Psychology Review* **27**, 318–326 (2007).

42. Daughters, S. B. *et al.* Effectiveness of a brief behavioral treatment for inner-city illicit drug users with elevated depressive symptoms: the life enhancement treatment for substance use (LETS Act!). *Journal of Clinical Psychiatry* **69**, 122 (2008).

43. Mazzucchelli, T., Kane, R. & Rees, C. Behavioral Activation Treatments for Depression in Adults: A Meta-analysis and Review. *Clinical Psychology: Science and Practice* **16**, 383–411 (2009).

44. Patel, V. *et al.* The Healthy Activity Program (HAP), a lay counsellor-delivered brief psychological treatment for severe depression, in primary care in India: a randomised controlled trial. *The Lancet* **389**, 176–185 (2017).

45. Van Ginneken, N. *et al.* Non-specialist health worker interventions for the care of mental, neurological and substance-abuse disorders in low-and middle-income countries. *Cochrane database of systematic reviews* (2013).

46. Crane, C. A., Eckhardt, C. I. & Schlauch, R. C. Motivational enhancement mitigates the effects of problematic alcohol use on treatment compliance among partner violent offenders: Results of a randomized clinical trial. *Journal of Consulting and Clinical Psychology* **83**, 689–695 (2015).

47. Arkowitz, H., Miller, W. & Rollnick, S. *Motivational interviewing in the treatment of psychological problems*. (Guilford Publications, 2015).

48. Balán, I. C., Lejuez, C. W., Hoffer, M. & Blanco, C. Integrating Motivational Interviewing and Brief Behavioral Activation Therapy: Theoretical and Practical Considerations. *Cognitive and Behavioral Practice* **23**, 205–220 (2016).

49. Pulerwitz, J. & Barker, G. Measuring Attitudes toward Gender Norms among Young Men in Brazil: Development and Psychometric Evaluation of the GEM Scale. *Men and Masculinities* **10**, 322–338 (2007).
50. Puffer, E. S. *et al.* Family Functioning and Mental Health Changes Following a Family Therapy Intervention in Kenya: a Pilot Trial. *Journal of Child and Family Studies* 1–16 (2020).
51. Essau, C. A., Sasagawa, S. & Frick, P. J. Psychometric properties of the Alabama parenting questionnaire. *Journal of Child and Family Studies* **15**, 595–614 (2006).
52. Puffer, E. S. *et al.* Development and Evaluation of the Family Togetherness Scale: A Diagnostic Accuracy Study.
53. QRS International Pty Ltd. *NVivo (Version 12)*. (2018).
54. Brown, R. L., & Rounds, L. A. (1995). Conjoint screening questionnaires for alcohol and other drug abuse: criterion validity in a primary care practice. Wisconsin medical journal, 94(3), 135–140.
55. Maina, Elizabeth & Wagoro, Miriam & Angeline, Kirui & Lincoln, Khasakhala. (2017). Perceived social support among clients using alcohol and illicit drugs at the comprehensive care center at the coast general hospital – Kenya: A descriptive cross-sectional study. International Journal of Advanced Research. 5. 1075-1082. 10.21474/IJAR01/5868.
